# Hydrogel-Based BMP-2 Delivery for Bone Regeneration: From Material Control to Biological and Translational Evaluation

**DOI:** 10.3390/polym18192349

**Published:** 2026-09-26

**Authors:** Tingting Zhou, Xian He, Jiahe Li, Xianglong Han, Jie Feng, Ye Tian

**Affiliations:** 1 State Key Laboratory of Oral Diseases & National Center for Stomatology & National Clinical Research Center for Oral Diseases, Department of Orthodontics, West China Hospital of Stomatology, Sichuan University, Chengdu 610041, China; 2024224035167@stu.scu.edu.cn (T.Z.); 2024224035181@stu.scu.edu.cn (X.H.); xhan@scu.edu.cn (X.H.); 2 State Key Laboratory of Oral Diseases & National Center for Stomatology & National Clinical Research Center for Oral Diseases, Department of Cariology and Endodontics, West China Hospital of Stomatology, Sichuan University, Chengdu 610041, China; jiaheli@stu.scu.edu.cn

**Keywords:** BMP-2, hydrogels, growth factor delivery, controlled release, bone regeneration, osteoinduction

## Abstract

Bone morphogenetic protein-2 (BMP-2) is a potent osteoinductive factor, but rapid loss of bioactivity, short local retention, burst release, and diffusion into surrounding tissues reduce dose efficiency and may necessitate supraphysiological doses associated with local adverse effects. Hydrogels offer tunable platforms for controlling BMP-2 localization, exposure, and presentation. This review classifies BMP-2-delivering hydrogels by their dominant delivery logic into passive spatial confinement, reservoir-unit-integrated delivery, stimuli-responsive delivery, and programmable biointeractive systems. It then evaluates whether material-level control translates into retained bioactivity, cellular responses, bone regeneration, spatially confined bone formation, and clinically relevant outcomes across conventional and complex-host models. Selected systems preserve BMP-2 activity after chemical modification, immobilization, or triggered release and improve bone formation, structural repair, or spatial localization in specific models. Nevertheless, prolonged retention or greater material complexity does not consistently improve repair, and evidence for true dose sparing remains limited. Outcomes depend on actual bioactive exposure, material degradation, tissue ingrowth, host conditions, and auxiliary components. Translation therefore requires formulation-specific links among dose, bioactive exposure, repair efficacy, local safety, and reproducible manufacturing.

## 1. Introduction

Repair of large bone defects, nonunion, and bone defects under unfavorable host conditions, such as infection or osteoporosis, remains a major clinical challenge in orthopedic and oral and maxillofacial surgery [1,2,3]. Bone morphogenetic protein-2 (BMP-2) has well-established osteoinductive activity and can promote the osteogenic differentiation of mesenchymal stem cells (MSCs) [4,5,6]. Recombinant human BMP-2 (rhBMP-2), delivered using an absorbable collagen sponge (ACS), has been developed as a clinical product and approved for selected bone repair indications [7,8,9]. Clinical use has demonstrated the potential of BMP-2 to promote bone formation, but has also revealed adverse effects associated with inadequate control of local delivery and the use of high doses [10,11,12,13].

Free BMP-2 is susceptible to degradation and is rapidly cleared after administration in vivo. ACS primarily loads rhBMP-2 through physical adsorption and provides limited control over protein release and local retention [14,15,16]. As a result, substantial burst release may occur during the early post-implantation period [17]. To maintain an adequate local osteogenic signal, clinically used BMP-2 doses are often far above physiological levels, which may increase the risk of ectopic bone formation, soft-tissue swelling, inflammation, and bone resorption [7]. The low mechanical strength of ACS also limits its ability to maintain space in complex bone defects [18]. These limitations highlight the need for delivery systems that preserve BMP-2 stability while providing better control over its release, local retention, and spatial distribution.

Hydrogels offer several advantages for BMP-2 delivery. Their highly hydrated three-dimensional structures mimic key features of the native extracellular matrix (ECM) and can provide a favorable environment for maintaining BMP-2 bioactivity [19,20,21,22]. Their hydrated polymer networks can restrict BMP-2 diffusion by regulating pore architecture, crosslinking density, and degradation behavior [23]. Hydrogels can also incorporate affinity components such as heparin, microsphere or nanoparticle reservoirs, stimuli-responsive linkages, and nucleic acid- or cell-mediated local expression systems. These design strategies can modulate the loading location, release resistance, duration of exposure, and source of BMP-2 signaling [15,24,25,26]. In addition, injectable hydrogels can conform to irregular defects, while in situ gelation helps retain the delivery system at the implantation site [27,28]. Taken together, these features position hydrogels as alternative local delivery platforms designed to address the major limitations of ACS-mediated BMP-2 delivery, including bioactivity loss, burst release, uncontrolled spatial diffusion, and dependence on supraphysiological doses (Figure 1).

However, protein loading and release profiles alone cannot determine whether BMP-2 remains biologically active after material processing [29]. Increased local retention also does not necessarily translate into greater biologically effective BMP-2 exposure or improved bone regeneration [15]. Therefore, despite the inherent advantages of hydrogels for BMP-2 loading and delivery, their therapeutic benefit must be established through a continuous chain of biological evidence. Evaluation of hydrogel-mediated BMP-2 delivery should extend beyond loading and release to BMP-2-specific bioactivity, bone formation, osseous bridging or spinal fusion, biomechanical performance, and the anatomical distribution of newly formed bone [30]. It is also necessary to determine how these outcomes are influenced by BMP-2 dose, material degradation, auxiliary components, and the host environment [19,31,32].

This review is organized around the delivery logic of BMP-2 hydrogels and whether material-level control can translate into meaningful bone repair benefits. Section 2 classifies these systems according to the principal mode by which the material controls BMP-2, including passive spatial confinement, reservoir-unit-integrated delivery, stimuli-responsive delivery, and programmable biointeractive systems. Section 3 further evaluates in vitro bioactivity and cellular responses after material processing and delivery, regenerative efficacy and the spatial distribution of bone formation in conventional bone repair models, and repair performance under complex host conditions. It also examines the evidence hierarchy of current models, their clinical relevance, and the limits of inference that can be drawn from them. Finally, we discuss future directions for BMP-2 hydrogel delivery, with particular attention to the reporting of modality-specific input doses, protein release, and local expression, therapeutic windows, attribution of effects within multifunctional systems, and considerations related to manufacturing and quality control.

## 2. Hydrogel-Based Strategies for BMP-2 Delivery

For preloaded BMP-2 protein, hydrogel-mediated regulation depends on how the protein is incorporated within the material and how its release, local transport, and presentation to surrounding cells are controlled [25,26]. Continuous hydrogel networks can restrict BMP-2 diffusion through their mesh size, crosslinking density, and degradation behavior, while affinity sites within the network can further enhance local retention [23,26,33]. BMP-2 release can be regulated by microsphere size and crosslinking [34] or by barrier-layer design in multilayer films [35]. These studies establish release-control principles at the carrier level. Hydrogel-based implementations include BMP-2/polymer nanocomplexes that themselves form a gel [36] and BMP-2-loaded mineral-coated microparticles embedded within a surrounding hydrogel matrix [37]. Stimulus-dependent release has been demonstrated in oxidation-sensitive multilayer films on PLGA scaffolds, where oxidative conditions increased BMP-2 release [38]. In hydrogel-based systems, enzyme-sensitive networks [39], pH-responsive reservoirs [40], oxidation-sensitive components [41], and external ultrasound [42] provide distinct means of regulating local growth-factor release or availability. Nucleic acids, engineered cells, and endogenous signal capture further alter the source of BMP-2 signaling, extending local delivery from the administration of preloaded protein to the local production, secretion, or enrichment of BMP-2 [43,44,45,46]. Hydrogel-mediated control of BMP-2 therefore progresses from restricting the diffusion and loss of preloaded protein, to regulating its release through independent reservoirs or defined triggers, and further to modifying the source and mode of local BMP-2 presentation through encoded information, living components, or endogenous signal capture. Accordingly, this section discusses four design strategies—passive spatial confinement, reservoir-unit-integrated delivery, stimuli-responsive delivery, and programmable biointeractive systems—and examines how different material architectures regulate the local retention, release, production, and presentation of BMP-2 (Figure 2).

### 2.1. Passive Spatial Confinement Systems

Passive spatial confinement modulates the diffusion path of BMP-2 through a continuous hydrogel network by controlling mesh size, crosslinking density, swelling, and degradation. Early studies of gelatin hydrogels linked BMP-2 release to material water content, crosslinking, and degradation [47], while polyphosphazene systems showed that side-chain structure, ionic interactions, and polymer properties can jointly regulate the release rate [48]. Subsequent studies further controlled this process through measurable network parameters. Transglutaminase-crosslinked poly(ethylene glycol) (PEG) hydrogels simultaneously modulated stiffness, arginine-glycine-aspartic acid (RGD) content, and degradable crosslinking sites, allowing BMP-2 exposure to be jointly influenced by network architecture and the degradation profile of the matrix [49]. Triple-helical recombinant collagen-based composite hydrogels provided a porous matrix for BMP-2 incorporation, with experimentally characterized swelling and degradation behavior [50]. Rapid thiol-ene click-crosslinked PEG hydrogels limited the early escape of rhBMP-2 through in situ network formation and low swelling [51]. Network architecture, polymer-protein interactions, swelling, and degradation provide complementary design variables for regulating BMP-2 transport.

Silk-based hydrogels illustrate how gel formation and composite composition can regulate BMP-2 delivery. Zhang et al. incorporated vascular endothelial growth factor 165 (VEGF_165_) and BMP-2 into silk solutions after sonication and before completion of gelation, providing physical encapsulation without exposing the growth factors directly to the sonication step [52]. Here, sonication initiates network formation rather than triggering subsequent protein release. Ma et al. incorporated poly(lactic acid) (PLA) or poly(lactic-co-glycolic acid) (PLGA) into PEG-silk formulations. Compared with the particle-free PEG-silk hydrogel, the PLA-containing formulation exhibited increased hydrophobicity and structural stability, reduced early BMP-2 release, and slower degradation [53]. These approaches regulate delivery through network formation and composite properties. In the latter system, BMP-2 was introduced into the PEG-containing phase during preparation; the formulation should therefore be distinguished from systems fabricated by embedding separately prepared BMP-2-loaded particles into a hydrogel.

Introducing affinity sites into a continuous hydrogel network can further enhance local retention through noncovalent interactions between BMP-2 and the material. Heparinized chitosan exploits the affinity of heparin for BMP-2 to reduce protein loss and attenuate the loss of BMP-2 activity during incubation at 37 °C, protease exposure, and challenge with an antagonistic protein [54]. Fucoidan-functionalized deacetylated β-chitin provides a sulfated polysaccharide binding interface. At day 4, cumulative BMP-2 release was approximately 72.5% from SDGC and 20.3% from its fucoidan-functionalized counterpart, FD-SDGC; release from FD-SDGC reached approximately 37.1% at day 14 [55]. An injectable carboxymethyl chitosan (CMCS)/PEG/HS hydrogel further combines heparan sulfate (HS) binding with matrix degradation, such that release is jointly controlled by affinity-mediated retention and dynamic changes in the continuous network [56]. Heparin-conjugated fibrin similarly integrates protein binding and local immobilization within an ECM-like matrix [57]. The short peptide D-BMP2b can also provide selective binding sites, extending affinity-based design beyond glycosaminoglycan-derived interfaces [58].

Adhesive surface chemistry provides another route to local protein immobilization. Wang et al. treated a silk fibroin hydrogel containing nano-hydroxyapatite and graphene oxide with dopamine, followed by incubation in a BMP-2 solution [59]. This approach introduces an adhesive interface for protein loading beyond simple physical enclosure within the network. The reported immobilization procedure does not by itself demonstrate a specific covalent linkage between BMP-2 and the matrix, and the osteogenic responses observed with this hybrid formulation may arise from both BMP-2 and the accompanying material components. These factors should be considered when distinguishing protein immobilization from the biological effects of the complete system.

These systems retain preloaded BMP-2 through the continuous hydrogel network and its associated binding interfaces. Network architecture, swelling, and degradation regulate protein transport, while affinity binding and adhesive interfaces contribute to local retention.

### 2.2. Reservoir-Unit-Integrated Delivery Systems

When BMP-2 is first loaded into independently engineered structural units and these units are subsequently immobilized within the defect by a hydrogel, the principal resistance to release no longer arises solely from the continuous hydrogel network. Microparticles, nanoparticles, mineralized interfaces, core-shell microspheres, or localized hydrogels within scaffold pores can provide distinct functions, including protein binding, degradation-mediated control, and diffusion resistance. The surrounding hydrogel can then serve as a conformable filler, retain the reservoir units at the target site, and provide a secondary diffusion barrier. Studies of recombinant collagen microspheres showed that particle size and crosslinking affected BMP-2 release [34]. Incorporating protein-loaded particles into a hydrogel introduces an additional level of material organization. For example, BMP-2-loaded gelatin microparticles were dispersed in bioprinted alginate constructs as a sustained-delivery system [60]. A hexahistidine-tagged T4 lysozyme-BMP-2 fusion protein (His6-T4L-BMP2) was directionally immobilized on nickel-chelated nitrilotriacetic acid (Ni-NTA) microparticles through His_6_-Ni^2+^-NTA coordination and subsequently incorporated into a gelatin/transglutaminase/tannic acid hydrogel. This configuration combines coordination-mediated BMP-2 binding with incorporation of the microparticles into a surrounding hydrogel network [61]. Mineral-coated microparticles immobilized BMP-2 through mineral binding and electrostatic adsorption, while a chitosan (CS)/PEG hydrogel restricted particle migration [37]. DSS6-Fibroplex incorporated BMP-2 into bone-targeting nanolipid complexes and combined these reservoirs with a printed scaffold-hydrogel construct, extending the function of the reservoir from sustained release to local spatial targeting [62].

The importance of loading location is further illustrated by silk-based composites. Cheng et al. loaded BMP-2 onto silk nanofibers, hydroxyapatite particles, or both components before assembling injectable composite hydrogels, obtaining different release profiles within the same material platform [63]. The hydroxyapatite-loaded configurations illustrate particulate reservoirs, whereas silk-associated loading reflects retention by the fibrous matrix. Lv et al. incorporated BMP-2-associated MgFe layered double hydroxide (LDH) nanosheets into a chitosan/silk fibroin hydrogel containing platelet-derived growth factor-BB (PDGF-BB). The system exhibited earlier PDGF-BB release and more sustained BMP-2 release, attributed to their different interactions with the network and nanosheets [64]. Its thermoresponsive gelation facilitates local hydrogel formation, whereas the differential growth-factor release reflects the loading arrangement and binding interactions. Deferoxamine in the Cheng system and PDGF-BB and bioactive ions in the Lv system also contribute biological functions, so the repair effects of these composites cannot be assigned solely to BMP-2 release kinetics.

The spatial organization of particles and hydrogels can also establish hierarchical release pathways. In a bilayer gelatin methacryloyl (GelMA) scaffold, BMP-2-loaded poly(lactic-co-glycolic acid) (PLGA) nanoparticles were incorporated into a highly methacrylated bone-like layer, whereas VEGF-loaded PLGA nanoparticles were placed in a less methacrylated vascular-like layer. The two factors were therefore stored in distinct particle populations and hydrogel layers, and their release was jointly influenced by particle degradation, gel density, and spatial location [65]. An alternative configuration involved filling the pores of a 3D-printed PLGA scaffold with a BMP-2-loaded four-arm poly(ethylene glycol) functionalized with o-phthalaldehyde (4armPEG-OPA)/gelatin hydrogel. The hydrogel degraded over approximately 3 weeks and mediated local protein release, whereas the PLGA scaffold degraded over approximately 5 months and maintained macroscopic porosity and structural support [66]. These two designs represent “particles within layered hydrogels” and “hydrogels within porous scaffolds,” respectively. Together, they illustrate the considerable flexibility available for coordinating release control, spatial localization, and mechanical support.

Diffusion barriers can even be established within individual microspheres. Bilayer oxidized sodium alginate-carboxymethyl chitosan microspheres used electrostatic adsorption to retain BMP-2 in the inner layer, while an outer porous polyelectrolyte membrane increased diffusion resistance. This architecture separated the protein-binding region from the release barrier within a core-shell structure [67].

Reservoir-unit integration increases the design flexibility of BMP-2 loading position, diffusion pathway, and spatial compartmentalization. The amount of BMP-2 introduced during fabrication, the actual loading content, release from the reservoir unit itself, and release from the complete composite system reflect different levels of material control. Reporting these parameters together can clarify how multiple structural layers collectively determine the BMP-2 release process.

### 2.3. Stimuli-Responsive Delivery Systems

Stimuli-responsive hydrogel designs can regulate local BMP-2 delivery through stimulus-induced gelation or changes in protein release and accessibility. In a thermosensitive mPEG-PLGA copolymer linked with 2,2′-bis(2-oxazoline) (BOX), body temperature induces in situ gelation, after which BMP-2 release accompanies hydrogel degradation [68]. Matrix metalloproteinase (MMP)-sensitive PEG hydrogels covalently tether rhBMP-2 and rhBMP-9 to the network, while enzyme-sensitive linkages and matrix degradation regulate the exposure of the immobilized proteins [69]. pH-responsive systems commonly use inorganic particles, metal-organic frameworks, or dynamic covalent bonds to alter reservoir stability. In a polyacrylamide/polydopamine/silk fibroin (PDS) hydrogel, deferoxamine (DFO) serves as an early-release component, whereas BMP-2 is encapsulated within CaCO_3_-mineralized zeolitic imidazolate framework-90 (ZIF-90). Acidic conditions promote sequential disassembly of the mineral coating and ZIF-90, creating a temporal separation between early DFO release and delayed BMP-2 release [70]. Metal-polyphenol network nanoparticles carrying immobilized BMP-2 undergo loosening of their coordination structure under acidic conditions and are locally retained by a hyaluronic acid hydrogel [40]. In the aldehyde-functionalized hyaluronic acid (HA-CHO)/GelMA system, acidic conditions promote cleavage of Schiff-base bonds and preferential vascular endothelial growth factor (VEGF) release, whereas BMP-2 is released more slowly from mesoporous SiO_2_ nanoparticles [71]. An alginate/hydroxyapatite (HAp)/poly(vinyl alcohol) (PVA) hydrogel containing BMP-2-loaded zeolitic imidazolate framework-8 (ZIF-8) nanoparticles enabled faster PDGF-BB release and more sustained BMP-2 release; pH-dependent release was evaluated using a model protein [72]. These systems combine responsive material components with distinct growth-factor loading sites to regulate local delivery.

Reactive oxygen species (ROS)-responsive systems commonly use oxidation-sensitive boronate ester bonds to alter network crosslinking or reservoir exposure. In PVA hydrogels crosslinked with the ROS-responsive boronic acid crosslinker TSPBA (PVA-TSPBA), elevated ROS levels cleave boronate ester bonds and promote the early release of epigallocatechin gallate (EGCG) nanoparticles. BMP-2@tannic acid (TA)-ZIF-8 within this system achieved an encapsulation efficiency of 89.4 ± 3.3%, and BMP-2 release was accelerated under H_2_O_2_ conditions while retaining an overall sustained-release profile [41]. Dynamic boronate ester-based hyaluronic acid-boronic acid-tannic acid-poloxamer composite hydrogels incorporating hydroxyapatite (HTF@HA) similarly respond to acidic and oxidative conditions. This system combines antioxidant and anti-inflammatory effects with sustained BMP-2 delivery [73]. These examples illustrate how responsiveness to oxidative conditions can be incorporated into hydrogel platforms that combine microenvironment modulation with BMP-2 delivery.

External energy input can provide further temporal and quantitative control over BMP-2 release. In a hydroxybutyl chitosan (HBC) thermosensitive hydrogel, polydopamine-modified Mg-CaCO_3_ microspheres encapsulated BMP-2 at a loading of 144 ng/mg microspheres, with an encapsulation efficiency of 72.01 ± 8.96%. Near-infrared (NIR) irradiation increased cumulative BMP-2 release at 14 days from 37.66 ± 3.39% to 49.16 ± 2.09%, indicating that photothermal conversion can modulate BMP-2 release from the microspheres and surrounding matrix [74]. Focused ultrasound (fUS) can also trigger retro-Diels-Alder de-crosslinking in Diels-Alder-crosslinked PEG hydrogels, with BMP-2 release increasing as the duration and number of ultrasound exposures increase [75].

### 2.4. Programmable Biointeractive Systems

Programmable biointeractive systems use encoded information, engineered living components, or endogenous signal capture to generate and enrich BMP-2 signals within the defect region. Gene-activated matrices incorporate BMP2 gene polyplexes into polylactide (PLA) particles and further integrate them into chitosan or platelet-rich plasma (PRP)-fibrin hydrogels. After release, the plasmid complexes transfect local cells and drive BMP-2 expression within the defect [76]. Engineered exosomes enriched with Bmp2 mRNA can be immobilized within GelMA hydrogels through CP05 peptide linkers. In this system, the GelMA formulation and CP05-mediated immobilization modulate vesicle retention and release, whereas BMP-2 is generated after intracellular translation of the delivered mRNA [77]. PEGylated poly(glycerol sebacate) acrylate (PEGS-A) hydrogels can also carry small extracellular vesicles (EVs) enriched with mRNAs encoding BMP-2 and VEGF-A, thereby delivering the coding information for both growth factors to local cells [78].

In these systems, the hydrogel regulates the local retention and release of nucleic acid complexes or EVs, whereas BMP-2 is produced by cells that receive the encoded information. These systems should be characterized by BMP-2 expression level, duration, and controllability, alongside measurements of nucleic acid or vesicle release.

Engineered cells and other living components further transform hydrogels into local sites of BMP-2 production. Human bone marrow-derived mesenchymal stem cells genetically engineered to express BMP-2 can be encapsulated within photocrosslinked hydrogels and continuously produce BMP-2 within the material. In this setting, local signal output is jointly determined by cell viability, transgene expression, and cell-material interactions [79]. Nitric oxide (NO)-sensing engineered bacteria can be encapsulated within GelMA microspheres and further embedded in hyaluronic acid methacryloyl (HAMA) hydrogels. These living components sense local NO signals and activate BMP-2 synthesis and secretion [45]. This design directly couples chemical cues in the tissue environment to BMP-2 production, allowing the living components within the hydrogel to modulate protein output according to local conditions.

In addition to delivering encoded information and engineered living components, material interfaces can capture endogenous BMP-2 generated during tissue repair. BMP-2-binding peptide (BBP)-modified tetrahedral framework nucleic acids (tFNAs) can be immobilized within HAMA hydrogels, where their multivalent binding interfaces enrich endogenous BMP-2 and restrict its local diffusion [46]. In B2A-functionalized gradient hydrogels, nanohydroxyapatite immobilized within the aldehyde-modified HAMA bone layer captures endogenous BMP-2. Grafted B2A enhances BMP-2-associated osteogenic responses in the bone layer and promotes chondrogenic responses in the cartilage layer [80].

Programmable biointeractive systems therefore organize local BMP-2 signaling through three principal modes: delivery of BMP2-encoding information, local BMP-2 production by engineered cells or living components, and enrichment of endogenous BMP-2 through material interfaces. In these systems, hydrogels provide functions such as nucleic acid or vesicle immobilization, cell encapsulation, and signal capture. BMP-2 delivery is thereby extended from the release of preloaded protein to the local generation and spatial organization of BMP-2 signaling. Representative hydrogel platforms across the four delivery-strategy classes discussed in Section 2, together with their BMP-2 modalities, dominant control mechanisms, release, production, or presentation characteristics, and evaluation models, are summarized in Table 1. Differences among BMP-2 modalities in onset, duration, administered input, controllability, and safety considerations are summarized in Table 2.

## 3. Biological Evaluation and Clinical Relevance

In systems containing preloaded BMP-2 protein, hydrogel-mediated regulation of protein loading, local retention, and release primarily reflects material-level control over the delivery process. Determining whether these changes translate into biological and therapeutic benefits requires assessment of whether BMP-2 retains its bioactivity after processing, material binding, or release, and whether it subsequently promotes the formation of structurally continuous new bone within the target defect [29,30,81]. Bone metabolic status, persistent inflammation, bacterial infection, and material-associated immune responses can all modify this process, causing the same delivery system to produce different outcomes under different host conditions [32,82,83,84]. The specific repair challenge represented by an animal model and the bone repair endpoints used for evaluation also determine how closely the resulting evidence relates to clinical application. Accordingly, this section evaluates BMP-2 bioactivity and bone repair outcomes in the context of the models in which they are assessed, and considers the clinical relevance of current evidence based on the conclusions that these studies can reasonably support.

### 3.1. In Vitro Bioactivity and Cellular Responses

For hydrogels containing preloaded BMP-2 protein, loading capacity and release profiles describe protein incorporation and release. Whether BMP-2 retains its biological activity after material processing requires further verification. Schoonraad et al. thiolated rhBMP-2 derived from Chinese hamster ovary (CHO) cells and observed no loss of activity in a SMAD1/5/8 reporter assay. After the thiolated BMP-2 was immobilized within an MMP-sensitive PEG hydrogel, 100 and 500 nM BMP-2 increased inhibitor of DNA binding 1 (ID1) expression in encapsulated human MSCs by 190% and 620%, respectively, compared with BMP-2-free hydrogels [69]. These findings indicate that hydrogel-immobilized BMP-2 can retain its ability to activate BMP-specific signaling. In another study, BMP-2 released from a focused ultrasound-triggered Diels-Alder PEG hydrogel was adjusted to 100 ng/mL and compared with soluble BMP-2 at the same concentration. No significant differences in alkaline phosphatase (ALP) activity or mineralization were detected between the two groups [75], suggesting that the release process did not substantially impair the capacity of BMP-2 to induce osteogenic responses. Together, these studies demonstrate at the levels of BMP-specific signaling and downstream osteogenic phenotypes that BMP-2 can retain its biological activity after chemical modification, material immobilization, or triggered release. This provides a biological basis for using hydrogels to regulate its local presentation and release.

Low cumulative BMP-2 release does not necessarily imply a weak cellular response. Heparin methacrylamide microparticles released less than 25% of their bound BMP-2 over 28 d, while 0.1 mg of microparticles loaded with 10 or 30 ng BMP-2 induced ALP activity normalized to deoxyribonucleic acid (DNA) content in C2C12 cells. In BMP-2-treated cultures, direct contact with the microparticles increased DNA content and total ALP activity relative to Transwell-separated cultures, whereas DNA-normalized ALP activity was similar [85]. In a separate study, a self-assembling peptide hydrogel loaded with 300 ng BMP-2 released approximately 0.4% over 12 d in a distilled-water release assay. In cell-culture experiments, BMP-2-loaded hydrogels placed in Transwell inserts increased ALP activity in cells cultured in the lower compartment [86]. These findings indicate that cumulative release alone is insufficient to predict cellular responses and should be considered together with BMP-2 bioactivity and cell-material interactions.

Cell adhesion provides an additional determinant of how cells interact with a BMP-2-delivering matrix. Kisiel et al. grafted the integrin-specific fibronectin fragment FNIII9*-10 onto hyaluronic acid hydrogels and demonstrated greater MSC attachment and spreading than on the corresponding unmodified hydrogels [87]. These findings establish an improvement in the cellular interaction with the carrier, rather than direct evidence of enhanced BMP-2 stability or slower release. Full-length fibronectin introduces a broader range of functions. Trujillo et al. incorporated full-length fibronectin into PEG hydrogels to support growth-factor sequestration and presentation [88]. Together, these studies distinguish modification of the cell-adhesive interface from regulation of growth-factor binding and availability. Both can influence the response to locally delivered BMP-2, but improved cell attachment should not itself be interpreted as proof that protein bioactivity has been preserved.

Affinity-based designs not only influence BMP-2 retention and release but can also slow the loss of protein activity under unfavorable conditions. After incubation at 37 °C or exposure to MMP-9, heparinized chitosan retained higher BMP-2 concentrations and greater ALP-inducing activity than phosphate-buffered saline (PBS) or non-heparinized chitosan. It also maintained a stronger response in the presence of the BMP antagonist noggin [54]. Fucoidan/poly-L-lysine (PLL) coacervate droplets similarly prolonged the ALP response of MC3T3-E1 cells and partially counteracted inhibition by noggin [89]. In fucoidan-modified chitin systems, soluble BMP-2 produced a stronger early ALP response, whereas the carrier group maintained a greater response at later culture stages [55]. Collectively, findings obtained under thermal stress, proteolytic degradation, and antagonistic conditions indicate that affinity materials can reduce the premature loss of BMP-2 functional activity and extend its effects beyond a short-lived stimulus.

Affinity interactions, however, are also constrained by binding strength. Excessively strong binding may increase BMP-2 retention while reducing its accessibility to cells. In maleimide-functionalized poly(ethylene glycol) (PEG-Mal) hydrogels grafted with BMP-2-specific affibodies, the high-affinity formulation achieved greater loading and released less BMP-2 in serum-containing medium. However, precomplexing the affibody with BMP-2 reduced the ALP response of C2C12 cells. In post-release bioactivity assays, the high-affinity group also induced the lowest ALP response [90]. Reducing protein loss is therefore not the sole objective of affinity-based delivery. BMP-2 retention must also preserve sufficient accessibility for cellular recognition and utilization. Affinity materials should therefore balance delayed loss of activity with maintenance of receptor accessibility.

The activity retained by hydrogel-delivered BMP-2 can further manifest as downstream responses, including cell migration, osteogenic differentiation, and matrix mineralization. Eluates from heparin-conjugated fibrin hydrogels induced osteocalcin expression and calcium deposition [57]. Hydrogels composed of the RADA-based self-assembling peptide bearing an RGD motif (RADA-RGD) and BMP-2 promoted cell proliferation, migration, ALP activity, and mineralization [91]. Yuan et al. developed bilayer oxidized sodium alginate-carboxymethyl chitosan microspheres that sustained BMP-2 release for 20 d. Compared with blank microspheres, BMP-2-loaded microspheres further enhanced rat bone marrow-derived mesenchymal stem cell migration and increased ALP activity, osteopontin (OPN)/osteocalcin (OCN) expression, and mineralization at 7, 14, and 21 d, respectively [67]. These studies extend the evidence that BMP-2 remains signaling-competent after material processing to downstream stages of osteogenic differentiation, osteogenic protein expression, and matrix mineralization. They indicate that hydrogel-delivered BMP-2 can induce osteogenesis-related responses across multiple stages in vitro.

Overall, current in vitro evidence supports that hydrogel-based delivery can preserve BMP-2 bioactivity while regulating its local presentation and duration of action. BMP-2 can retain the ability to activate specific signaling pathways after chemical modification, material immobilization, or triggered release. Hydrogel-immobilized BMP-2 can also retain biological activity. Affinity components such as heparin and fucoidan can further prolong BMP-2 functional activity under degradative or antagonistic conditions. The resulting cellular responses span multiple levels, including BMP-specific signaling, ALP activity, osteogenic protein expression, and mineralization. These findings indicate that material-level control of BMP-2 can translate into measurable biological responses in vitro.

### 3.2. Regenerative Efficacy and Spatial Distribution of Bone Formation in Conventional Models

The in vivo value of hydrogel-mediated BMP-2 delivery is reflected mainly in three aspects: whether improved bone repair can be achieved at the same BMP-2 dose, whether carrier design can reduce the BMP-2 dose required for effective repair, and whether newly formed bone can be preferentially confined to the target region. In this section, conventional bone repair models refer to bone defect and fusion models without additional pathological conditions such as infection, osteoporosis, ischemia, or persistent inflammation.

Carrier comparisons at the same BMP-2 dose can directly reveal the effect of the complete delivery platform on bone repair. Zhou et al. delivered 0.5 μg BMP-2 using a gelatin sponge, a CMCS/PEG hydrogel, or a CMCS/PEG hydrogel containing HS. The HS-containing system released approximately 15% of BMP-2 on the first day, whereas the hydrogel without HS released more than 40%. At 6 weeks, bone volume/total volume (BV/TV) reached 52.05% in the HS group and was higher than that in the HS-free group [56]. These findings link affinity-mediated regulation of BMP-2 release with increased bone formation within the defect. Kitahara et al. compared hydroxyapatite (HAp)/beta-tricalcium phosphate (β-TCP) microsphere-hydrogel constructs and collagen sponges, each loaded with 10 μg rhBMP-2, in a rat femoral nonunion model. At 6 weeks, osseous union was observed in 13/17 defects in the microsphere-hydrogel group and 6/17 defects in the collagen sponge group. The corresponding new bone areas were 56.2% and 40.7%, respectively (*p* = 0.0284) [92]. Unlike a simple increase in radiographic bone volume, osseous union indicates the formation of continuous new bone bridging the two ends of the defect. Although these studies used different models and endpoints, both showed effects in the same direction. Together, they suggest that integrating affinity components or reservoir units with hydrogels can improve overall repair performance without increasing the administered BMP-2 dose.

The relationship between BMP-2 dose and repair outcome provides the basis for evaluating dose efficiency. Peng et al. used the thermosensitive BOX hydrogel to deliver 5, 10, or 20 μg rhBMP-2 in a 10 mm rabbit femoral defect model. At 12 weeks, healing occurred in 2/6, 4/6, and 6/6 defects, respectively, whereas no healing was observed in either the untreated group or the BMP-2-free hydrogel group. Complete bridging was achieved in the 20 μg group, and both torsional stiffness and maximum torque were comparable to or greater than those in the autologous bone graft group [68]. This study shows that increasing the BMP-2 dose within the same hydrogel platform progressively improved the bridging rate and mechanical recovery. A comparable dose-dependent pattern was reported by Lienemann et al. in 4 mm critical-sized mouse calvarial defects: 0.2 μg BMP-2 produced limited healing, whereas 0.5 and 1.0 μg resulted in nearly complete defect coverage, although bone volume continued to increase approximately linearly with dose [39] (Figure 3). Both studies define dose-response relationships within specific hydrogel platforms, but neither directly demonstrates that the hydrogel reduces the BMP-2 dose required for effective repair. In a rat caudal vertebral fusion study, collagen carriers containing 3 or 10 μg BMP-2 achieved fusion rates of 50% and 62.5%, respectively, whereas HAp/β-TCP/hydrogel constructs achieved 87.5% fusion at both doses [93]. This finding shows that changing the carrier alone can improve fusion at the same 3 μg BMP-2 dose. In another study, a supramolecular hydrogel containing 1.5 μg BMP-2 achieved repair outcomes approaching those obtained with a collagen carrier containing 15 μg BMP-2 [58], providing evidence of potentially greater repair efficiency at a lower dose across different carriers and dose levels. These four studies address three distinct aspects of dose efficiency. The rabbit femoral and mouse calvarial defect studies define dose-response relationships within individual hydrogel platforms. The caudal fusion study compares carrier performance at the same BMP-2 dose, whereas the supramolecular system examines whether a lower-dose platform can approach the repair performance of a higher-dose reference carrier. Taken together, these findings indicate that dose efficiency should not be defined simply as a reduction in the amount of BMP-2 administered. Rather, it refers to the ability to maintain bone bridging, fusion, or mechanical recovery at a lower BMP-2 dose.

In a minipig lumbar fusion model that more closely approximates clinical surgical conditions, increasing the BMP-2 dose did not produce a sustained increase in fusion benefit. Fifty-kilogram minipigs underwent lateral lumbar interbody fusion with polyetheretherketone (PEEK) cages and internal fixation, while the same HAp/β-TCP/hydrogel platform delivered 0, 500, or 1000 μg BMP-2. Both the 500 and 1000 μg groups showed enhanced interbody fusion, but the 1000 μg group achieved no additional fusion benefit. Instead, bone spurs extending toward the spinal canal were observed at 8 and 16 weeks, together with local fibrosis and inflammation [94]. These findings indicate that once the BMP-2 dose is sufficient to support fusion, further dose escalation may preferentially expand the spatial extent of bone formation and local tissue responses rather than improve fusion itself. Dose efficiency in hydrogel-mediated delivery therefore also involves defining an appropriate dose range that balances fusion efficacy against the spatial extent of bone formation. The spinal canal-directed bone spurs observed in the minipig model further highlight the importance of evaluating where newly formed bone develops.

In an 8 mm rat femoral defect model, delivery of 30 μg BMP-2 alone resulted in 65.8 ± 5.3% of the total new bone forming outside the defect at 4 weeks. After incorporation of 0.1 or 1 mg heparin microparticles, this proportion decreased to 39.4 ± 5.9% and 44.8 ± 7.9%, respectively, while no significant differences were detected in bone bridging, mineral density, or torsional load at 12 weeks [15]. The advantage of the heparin microparticles was therefore not reflected by greater final bone volume or mechanical performance, but by a greater proportion of new bone being confined to the target defect. In another study, a rapidly click-crosslinked PEG hydrogel and a collagen carrier were used to deliver the same 1 μg dose of BMP-2. Both promoted calvarial defect closure, but the collagen group produced more bone outside the defect. In contrast, ectopic bone formation in the PEG hydrogel group did not differ significantly from that in the BMP-2-free control group [95]. Approximately 87% of BMP-2 was released from this PEG hydrogel in vitro by day 2, indicating that spatial restriction of bone formation does not necessarily depend on prolonged sustained release.

Silk hydrogel delivery in segmental long-bone defects further illustrates the distinction between release behavior and functional repair. Diab et al. delivered BMP-2 using silk hydrogels within polycaprolactone (PCL) nanofiber mesh tubes in an 8 mm rat femoral defect model and evaluated bone formation, bridging, and torsional properties [96]. Although the two silk concentrations produced different in vitro BMP-2 release profiles, no significant differences were detected between the corresponding BMP-2-containing groups in the measured in vivo outcomes. When the concentration groups were pooled, BMP-2 supplementation improved bone formation and biomechanical properties relative to silk without BMP-2. The findings support the regenerative potential of the combined delivery construct while showing that the observed release differences did not establish a detectable repair advantage. Interpretation also depends on the supporting mesh tube, fixation, and limited statistical power of the study.

These positive findings do not imply that strengthening any single parameter of material control will continuously improve therapeutic efficacy or optimize bone repair outcomes. A mineralized microparticle composite system showed an osteogenic benefit from BMP-2 supplementation at 4 weeks, but no significant difference from the BMP-2-free mineralized microparticle group was detected at 8 weeks [37]. This suggests that accelerated early bone formation does not necessarily result in a higher final level of repair. In the study by Hettiaratchi et al., heparin microparticles reduced cumulative BMP-2 release over 21 d by approximately 45% and increased early in vivo retention. However, at 12 weeks, bone bridging was achieved in 5/5 defects in the BMP-2-alone group but in only 3/5 defects in the microparticle-loaded BMP-2 group. Overall bone formation was also greater in the former group (*p* < 0.001) [33]. These two studies illustrate from different perspectives that improvements in intermediate delivery parameters cannot substitute for final bone repair outcomes. Slower release and greater local retention can translate into bone healing only when they generate BMP-2 signals that remain accessible to cells over an appropriate time window.

A study of PEG hydrogels further showed that network stiffness and matrix metalloproteinase (MMP)-mediated degradability jointly influenced the location and extent of bone formation. Simply reducing stiffness did not consistently increase new bone formation because the effect of degradability also varied with the mechanical context of the hydrogel [49]. Material degradation therefore does not act independently of network architecture. Within a given mechanical environment, it simultaneously affects cell infiltration, BMP-2 accessibility, and the space available for new bone formation. Another study using a degradation-responsive system further demonstrated this interaction. With the same PEG/RGD backbone and a 5 μg BMP-2 dose, the highly MMP-sensitive hydrogel achieved bone bridging in 6/6 defects at 4 weeks, supporting the view that network degradation can facilitate tissue infiltration and new bone formation [19].

A cell-mediated degradation system also improved bone repair in a rabbit radial defect model [97], whereas a fucoidan-modified chitin system sequentially linked BMP-2 affinity binding, sustained release, preservation of bioactivity, and in vivo bone formation within the same platform [55]. The former systems create space for tissue ingrowth through cell-mediated changes in the network, whereas the affinity-modified system simultaneously regulates protein retention and preservation of activity. Although these approaches target different material processes, they collectively indicate that the role of hydrogels should extend beyond delaying BMP-2 diffusion. Protein presentation, material degradation, and tissue regeneration need to be coordinated in both time and space.

Coordination with angiogenic signals can also influence the outcome of silk-mediated BMP-2 delivery. In the rabbit maxillary sinus augmentation study by Zhang et al., combined VEGF165/BMP-2 delivery increased new bone formation compared with BMP-2 alone and was accompanied by greater vascularization and faster loss of residual gel [52]. However, the elevated sinus floor height did not differ significantly between the combined and BMP-2-only groups at 12 weeks. Thus, the benefit depended on the selected endpoint: improved bone formation did not yield a corresponding increase in augmentation height. This study supports evaluating vascularization, material degradation, and anatomical repair objectives separately when interpreting the effects of combined delivery.

This coordination can also extend to inflammatory and oxidative stress responses during the early stages of defect repair. Wan et al. combined early aspirin release with near-infrared-triggered BMP-2 release to regulate the local environment at different stages of healing in healthy rat calvarial defects. Aspirin reduced inflammation-related markers, including tartrate-resistant acid phosphatase (TRAP), MMP-9, cyclooxygenase-2 (COX-2), and CD11b, within 48 h after implantation. At 8 weeks, BV/TV reached 55.20% in the complete combination group, compared with 39.86% in the BMP-2 module group [74]. Another study used sequential delivery of catalase (CAT) and BMP-2. CAT reduced local ROS levels during the early repair phase, followed by sustained BMP-2 presentation from CaCO_3_ microspheres. At 2 weeks, CAT-containing systems showed lower CD80 and higher CD206 signals, and the combined CAT/BMP-2 group achieved a BV/TV of approximately 70% at 8 weeks [98]. These studies link early modulation of inflammation or oxidative stress with subsequent bone formation. They indicate that, even in conventional defects, hydrogel design can extend beyond control of BMP-2 release and material degradation to coordinate the timing of auxiliary components with BMP-2 according to the progression of bone repair.

Previous studies have evaluated BMP-2-loaded hydrogels using bone volume, bone bridging or fusion, and mechanical performance. These endpoints reflect, respectively, the amount of new bone formation, structural continuity, and the functional quality of the repaired tissue. Current evidence indicates that some hydrogel platforms can improve bone formation and structural repair at the same BMP-2 dose and can better confine new bone to the target region. Effective repair achieved at lower doses also suggests the potential for improved dose efficiency. The in vivo value of hydrogel-mediated BMP-2 delivery therefore does not depend on release duration or material complexity per se. Rather, it depends on whether BMP-2 signaling is appropriately coordinated with material degradation, tissue infiltration, and the progression of bone regeneration.

### 3.3. Effects of Complex Host Environments on BMP-2-Mediated Bone Repair

Osteoporosis, inflammation and oxidative stress, active infection, and material-associated host immune responses can alter bone repair through imbalanced bone remodeling, persistent tissue injury, or abnormal immune activation. The therapeutic performance of BMP-2-loaded hydrogels may therefore vary with host conditions. This section examines bone repair outcomes under these pathological or immunological backgrounds.

Bone repair under osteoporotic conditions depends on the balance between new bone formation and ongoing bone resorption. Xiao et al. co-delivered BMP-2 and EGCG in 5 mm calvarial defects in ovariectomized rats. EGCG was used to modulate early ROS and inflammation, while BMP-2 was released in a sustained manner. At 6 weeks, the complete platform achieved a new bone volume fraction of 55.1 ± 3.1%, together with improvements in osteogenic, vascular, and immune-related indicators [41]. This finding suggests that improving the local microenvironment can enhance BMP-2-supported bone repair under conditions of elevated oxidative stress and abnormal bone remodeling. The benefits of individual auxiliary modules, however, need to be interpreted according to specific bone-related endpoints. In femoral defects in aged ovariectomized mice, the BMP-2-only group achieved the highest trabecular number (Tb.N, from 1.362 to 5.657 mm^−1^) and the lowest trabecular separation (Tb.Sp, from approximately 0.87 to 0.44 mm), whereas the combination group showed higher bone volume fraction and bone mineral density (BMD) [40]. These findings indicate that antioxidant or antiresorptive modules can alter both net bone gain and repair architecture in osteoporotic defects. However, the advantages of combination therapy may differ across endpoints. New bone formation, bone resorption, and bone microarchitecture should therefore be evaluated separately.

Diabetes, periodontitis, and peri-implantitis have distinct etiologies, but all involve persistent inflammation or oxidative stress. These models can therefore be used to determine whether improving the local environment also enhances BMP-2-associated bone repair. Li et al. compared a blank hydrogel, an interleukin-10 (IL-10)-only module, a BMP-2-only module, and a combined delivery system in calvarial defects in diabetic rats with fluctuating blood glucose levels. At 8 weeks, the combined group achieved 97.2% osseous connectivity and greater bone formation than either single-module group. These outcomes were accompanied by improvements in early inflammation- and oxidative stress-related indicators [99]. Another study of diabetic femoral condyle defects compared a microenvironment-regulating module, a VEGF/BMP-2 module, and the complete combined platform. The complete platform produced more new bone at 4 weeks, further supporting the coordinated use of antioxidant microenvironment modulation with angiogenic and osteogenic signals [100]. Oral inflammatory models provide evidence from different anatomical sites. In periodontitis-associated maxillary defects, delivery of BMP-2 using an HTF@HA/concentrated growth factor (CGF) hydrogel increased bone volume fraction at 8 weeks and was accompanied by changes in inducible nitric oxide synthase and CD206 signals [73]. A periodontal platform integrating antibacterial treatment, photothermal therapy, and BMP-2 delivery likewise improved both inflammation-related indicators and alveolar bone repair [101]. In bacteria-induced peri-implantitis, a GelMA platform combining ultrasound-mediated antibacterial treatment, metformin, and BMP-2 delivery reduced local interleukin-6 (IL-6) and tumor necrosis factor-alpha (TNF-α) levels and decreased peri-implant bone loss [102]. Collectively, these studies indicate that simply increasing osteogenic signaling may be insufficient for complete repair in environments characterized by persistent inflammation or oxidative stress. Concurrently reducing inflammation, oxidative stress, or bacteria-related tissue injury can create more favorable conditions for BMP-2-supported bone formation.

When active infection is present within a defect, control of the bacterial burden has a direct impact on BMP-2-induced bone repair. Johnson et al. used a four-arm maleimide-functionalized poly(ethylene glycol) (PEG-4MAL) hydrogel to co-deliver 100 ng BMP-2 and 1 U lysostaphin (LST) in a Staphylococcus aureus-infected segmental radial defect model in mice. Infected defects without effective antibacterial treatment showed no new bone formation over 8 weeks. Co-delivery of BMP-2 and LST markedly increased new bone formation and osseous bridging at both 4 and 8 weeks, and torque and stiffness at 8 weeks were also higher than those in the untreated infected group [32]. Karyagina et al. similarly found that infected mice without LST formed no new bone in a calvarial defect model, whereas all LST-treated animals developed newly mineralized bone [103]. By 4 weeks, bacteria could no longer be cultured from most animals in the infected group that had not received LST, yet bone formation remained impaired. This finding suggests that inflammation and tissue damage caused by early infection can continue to compromise subsequent repair even after the bacterial burden declines. Antibacterial ion/BMP-2 platforms in infected rabbit femoral defects and antibacterial hydrogels delivering BMP-2-derived peptides also improved infection-related indicators and increased later bone formation [104,105]. Across these models, active infection can block BMP-2-supported bone regeneration. Timely reduction in the local bacterial burden and mitigation of infection-induced tissue damage can help restore subsequent new bone formation, osseous bridging, and mechanical function.

Pre-existing immune sensitization to hydrogel materials can also alter BMP-2-induced bone formation, even in the absence of ongoing infection. Isaac et al. implanted PEG microporous annealed particle (MAP) hydrogels with identical compositions and the same 500 ng BMP-2 dose into PEG-naive and PEG-sensitized mice. At 3 weeks, bone volume in sensitized male and female mice was 1.71- and 1.44-fold that of their respective naive controls, respectively, and collagen deposition was also increased. Despite the greater amount and thickness of newly formed bone, Masson’s trichrome staining revealed a more porous architecture and less organized collagen structure in sensitized animals, particularly in male mice (Figure 4). These changes were accompanied by anti-PEG antibodies and altered local immune-cell responses [106]. Because the BMP-2 dose and hydrogel composition were held constant while host sensitization status was experimentally varied, this study provides direct evidence within this model that material-specific host immunity can affect both the quantity and morphology of newly formed bone. Evaluation under complex host conditions should therefore distinguish bone quantity from tissue quality.

Current evidence indicates that complex host environments can modify the performance of BMP-2-loaded hydrogels at multiple stages of bone repair. In osteoporosis, the final outcome reflects the net balance between new bone formation and persistent bone resorption. Sustained inflammation and infection can disrupt the local conditions required for BMP-2-mediated osteogenesis. Material-specific host immunity may also cause increases in bone quantity to become uncoupled from improvements in tissue quality. Auxiliary modules with anti-inflammatory, antibacterial, or antiresorptive functions can address these host-related barriers and improve repair outcomes. Their value, however, should ultimately be judged by the formation of stable bone architecture and restoration of function.

### 3.4. Evidence Hierarchy, Clinical Relevance, and Limits of Inference

Progression of a BMP-2 hydrogel study from in vitro experiments to small-animal, large-animal, and even human studies does not mean that the strength of evidence automatically increases at each stage. Assessing clinical significance requires two questions to be addressed simultaneously: whether the model reproduces the key conditions of the intended indication, and to what extent the study design allows the observed outcomes to be attributed to the delivery system [107,108,109]. The former depends on factors such as defect site, loading conditions, host disease, surgical procedure, and follow-up duration [108,110]. The latter depends on dose and carrier controls, component-level comparisons, and the selected outcome measures [111]. A model may closely resemble a clinical procedure yet still fail to isolate the independent contribution of hydrogel-mediated delivery. Conversely, a well-controlled in vitro or small-animal study may clearly define a material effect but cannot directly represent therapeutic efficacy in patients. The key question is therefore whether model-indication matching and confidence in causal interpretation improve in parallel as the platform advances toward translation [112,113].

The relevance of a model to the intended indication is first reflected by the repair objective that it evaluates. Calvarial defect models primarily assess new bone formation and the spatial distribution of bone within and outside the defect. Segmental long-bone defects additionally require evaluation of osseous bridging and biomechanical performance, whereas spinal fusion models determine whether stable continuity can be established between adjacent vertebrae [56,68,92,93,95]. Models incorporating osteoporosis, persistent inflammation and oxidative stress, active infection, or material-related immune responses further introduce bone remodeling, inflammatory status, bacterial burden, and tissue quality into the evaluation [32,41,99,106]. The inclusion of interbody cages, internal fixation, and oral implantation procedures also makes delivery volume, material fixation, interface integration, and responses of adjacent anatomical structures part of the therapeutic assessment [94,114]. These models provide different forms of clinically relevant information and cannot be arranged into a single hierarchy based solely on animal size or disease complexity. Their value ultimately depends on whether they capture the key conditions of the clinical problem that the platform is intended to address.

Even when an appropriate model is selected, a positive result does not necessarily allow the effect to be attributed specifically to BMP-2 delivery. The choice of endpoint determines what a positive finding can actually demonstrate. BMP-specific signaling assays and comparisons between released BMP-2 and soluble BMP-2 at the same concentration can directly assess whether material processing alters BMP-2 bioactivity. In contrast, ALP activity, osteogenic gene expression, and mineralization reflect cellular responses to the material system as a whole [57,69,75,91]. Control design further determines which questions can be answered in animal studies. Comparing different carriers at the same BMP-2 dose can identify differences in therapeutic performance between delivery platforms [92,93,97]. Establishing a dose gradient within the same hydrogel can define the relationship between BMP-2 dose and bone repair outcomes [68]. Material-only and single-module controls can help distinguish the contributions of BMP-2, auxiliary treatments, and the hydrogel itself [32,95,99]. Thus, studies described as “effective” may differ substantially in evidential strength. Some show only that a combined system induces osteogenesis-related cellular responses, whereas others demonstrate that a specific carrier improves bone repair at the same BMP-2 dose.

As studies progress to rabbits, dogs, and minipigs, clinical relevance and the ability to attribute outcomes may begin to diverge. Larger defect and surgical scales allow for the assessment of material fixation, tissue maturation, interface integration, and responses of adjacent structures. However, some studies simultaneously modify the BMP-2 dose, bone marrow aspirate, or other therapeutic components, making the independent contribution of the hydrogel more difficult to isolate [114,115]. In contrast, the minipig lumbar fusion study retained a dose comparison within the same platform and evaluated bone formation near the spinal canal under conditions involving an interbody cage and internal fixation. It therefore strengthened both model relevance and the interpretation of dose-related effects [94]. This comparison illustrates that increasing animal size and surgical complexity broadens the range of clinically relevant questions that can be examined, whereas appropriate controls and indication-specific endpoints determine the strength of the conclusions that can be drawn.

Human studies are the closest to actual treatment, but they also make it most difficult to isolate the independent effect of the hydrogel from the overall surgical procedure. In a randomized clinical study involving 12 patients, a hyaluronan-based hydrogel with or without BMP-2 was evaluated in cranial defects after neurosurgery. Both hydrogel treatments improved healing relative to the negative control, emphasizing the contribution of the carrier to the observed repair outcome [116]. Clinical studies have also evaluated specific Escherichia coli-derived recombinant human BMP-2/HAp- or β-TCP-based hydrogel composite products for spinal fusion. A prospective exploratory study enrolled 20 adults with spinal deformity, and all 18 patients who completed 12 months of follow-up achieved radiographic fusion. This indicates that the composite product has been used in complex spinal surgery and evaluated clinically for 1 year [117]. However, the study lacked a concurrent control group, so the fusion outcome reflects the combined effects of the composite product, interbody cage, internal fixation, and the overall surgical procedure. A subsequent retrospective comparative study of 254 patients included a local autograft control group. Dynamic radiographic fusion rates per spinal level were 92.31% in the hydrogel composite group and 82.35% in the local autograft-only group, whereas computed tomography (CT)-based fusion rates and patient-reported functional outcomes did not show corresponding differences [118]. The active comparator provides additional information on relative efficacy, but non-randomized group allocation and concomitant use of local autograft still limit attribution of the observed effects to the hydrogel itself. These spinal fusion studies support the feasibility and preliminary efficacy of the evaluated product family, but do not justify extrapolation to other hydrogel architectures, BMP-2 sources, or bone defect indications.

Among the studies discussed in this review, evidence for material control, bioactivity, animal repair, and clinical feasibility is distributed across different platforms. These studies do not provide a common basis for ranking hydrogel designs across all levels of evaluation. The conclusion currently supported by the evidence is that several specific platforms improve bone repair in defined models or indications. Representative in vivo and clinically relevant studies are compared in Table 3 according to model context, key dose/comparator design, regenerative and spatial outcomes, and the resulting limits of inference. Hydrogel categories cannot yet be ranked uniformly according to material complexity or model hierarchy.

## 4. Current Challenges and Future Perspectives

Hydrogels have advanced BMP-2 delivery from simple protein encapsulation toward multilayered control of local signaling. Strategies including protein confinement, affinity binding, reservoir integration, stimuli-responsive release, and local signal generation can alter the loading, retention, and presentation of BMP-2. Some platforms have also preserved BMP-2 bioactivity in vitro and improved bone formation, bony bridging, or fusion in bone repair models, while promoting a more favorable spatial distribution of newly formed bone within the target region. These advances have also changed how hydrogel performance should be evaluated. Prolonged release, increased local retention, or greater material functionality indicates stronger control over the delivery process but does not by itself establish therapeutic value. The clinical benefit of material design depends on whether such control produces an appropriate BMP-2 exposure profile and ultimately translates into stable bone repair. A major task for future research is therefore to establish a continuous relationship between material parameters and therapeutic outcomes, so that BMP-2 presentation, material degradation, tissue infiltration, and bone regeneration are appropriately coordinated for specific indications.

Establishing this relationship first requires an accurate description of effective BMP-2 exposure. The amount added during fabrication, actual loading content, dose delivered per defect, and cumulative release fraction represent different stages of the formulation process, and no single parameter can fully describe the amount of biologically active BMP-2 encountered by the tissue. For hydrogels containing microparticles or nanoparticles, measuring release from the internal reservoir alone is also insufficient to represent the release behavior of the complete implanted system [7,119]. For systems containing preloaded BMP-2 protein, studies should document the protein source, formulation input, actual loading, released amount, residual material-associated BMP-2, and process-related losses. BMP-specific signaling assays or potency assays should then be used to confirm the functional activity of released BMP-2, thereby linking formulation dose to biologically active exposure. When BMP-2 is generated locally through nucleic acids, engineered cells, or other living systems, the same issue takes the form of expression level, duration, and controllability. In these settings, local signaling should be defined by expression kinetics rather than by the mass dose and release fraction used for preloaded protein. Regardless of whether BMP-2 is preloaded or generated locally, biologically active exposure should ultimately be related to new bone formation, bony bridging, interbody fusion, and relevant biomechanical performance. Bone formation outside the target region, osteophyte formation, and responses of adjacent tissues should be assessed in parallel. Such a “formulation input-bioactive exposure-therapeutic outcome” evidence chain is required to define a therapeutic window that balances repair efficacy and local safety for a specific platform and indication [8].

Even when BMP-2 exposure is appropriately controlled, the host environment can still determine whether the signal produces stable new bone. Persistent bone resorption in osteoporosis, chronic inflammation and oxidative stress, active infection, and material-related immune responses can all alter bone remodeling, cellular responses, and tissue repair. Multifunctional hydrogels should therefore identify and address the major repair barriers associated with a specific indication rather than simply incorporate additional components. The necessity of anti-inflammatory, antibacterial, antioxidant, antiresorptive, or proangiogenic modules should be determined by comparing BMP-2-only modules, auxiliary-component-only modules, and the complete system. When sequential delivery is proposed, it should also be compared with simultaneous release of the same components to determine whether temporal sequencing provides additional repair benefit [120]. This host-barrier-oriented design strategy can align material functions with the clinical problem and allow components without a meaningful therapeutic benefit to be eliminated experimentally. Each additional layer of platform complexity should therefore be justified by a measurable therapeutic gain.

Polymeric gene carriers offer structural tunability but require control of cytocompatibility and batch consistency [121]. Lipid nanoparticles (LNPs) provide flexible nucleic acid formulations, with challenges in intracellular delivery and stability. Adeno-associated viruses (AAVs) support persistent expression, with immune responses and repeat dosing as key limitations. EVs carry functional nucleic acids, with cargo heterogeneity, purification, and potency standardization complicating translation [122]. Hydrogels function as local depots and tissue-engineering scaffolds, supporting defect conformity, retention, and BMP-2 release or presentation. Their combination with gene carriers integrates local retention with intracellular delivery, as illustrated by BMP-2 mRNA-carrying EV-hydrogel systems for bone regeneration [77,78]. Platform selection should balance these delivery benefits against formulation and manufacturing complexity.

Platform selection must be translated into a final formulation that can be manufactured reproducibly and used reliably. Batch-to-batch variation in natural materials, degree of chemical modification, crosslinking density, uniformity of BMP-2 loading, and sterilization and storage conditions can all affect gelation, degradation, release behavior, and protein bioactivity. Particle-containing composites, dynamically crosslinked systems, stimuli-responsive platforms, and nucleic acid- or cell-mediated systems introduce additional quality attributes, including particle size distribution, trigger thresholds, duration of expression, termination mechanisms, and long-term biosafety [119,123,124]. These factors should be defined early in development together with administration volume, gelation time, and implantation or injection procedures and should be verified in the final formulation rather than assessed only in individual materials or intermediate components. Organizing material design, dose selection, efficacy evaluation, and manufacturing control around a specific bone defect or fusion indication may help translate positive experimental findings into reproducible therapeutic strategies. Hydrogels have substantially expanded the design space for local BMP-2 delivery, but they have not yet broadly eliminated dose-related risks or barriers to clinical application. Further progress will depend on whether material-level control, biological efficacy, local safety, and manufacturability can be continuously validated within the same platform.

## Figures and Tables

**Figure 1:**
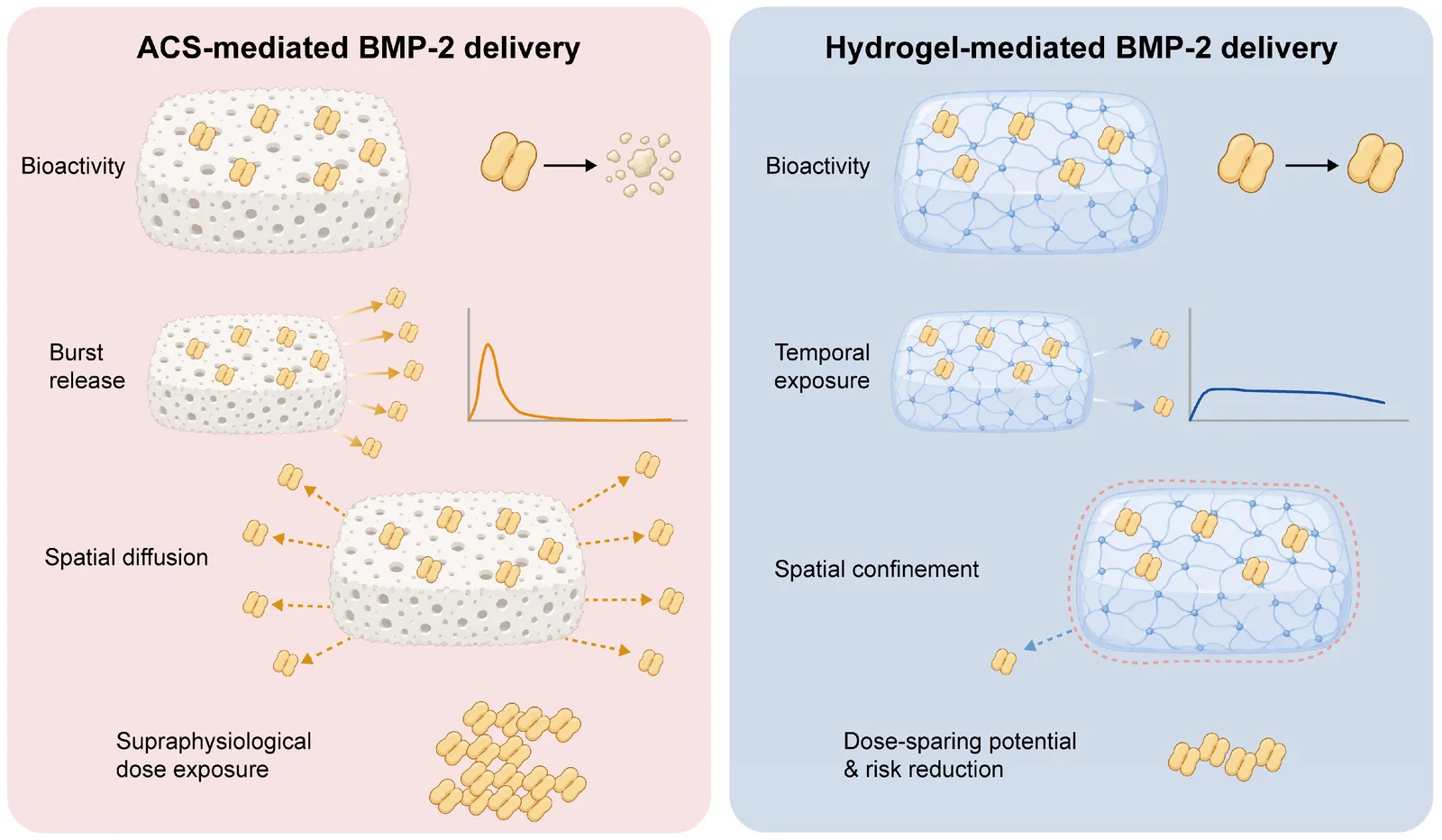
Conceptual comparison of ACS-mediated and hydrogel-mediated BMP-2 delivery. Absorbable collagen sponge (ACS)-mediated delivery may be associated with loss of BMP-2 bioactivity, early burst release, uncontrolled spatial diffusion, and dependence on supraphysiological doses. Hydrogel platforms are designed to protect BMP-2 bioactivity, regulate temporal exposure, improve spatial confinement, and potentially reduce the required dose and associated risks. The illustration is conceptual and does not imply that all hydrogel systems achieve these outcomes.

**Figure 2:**
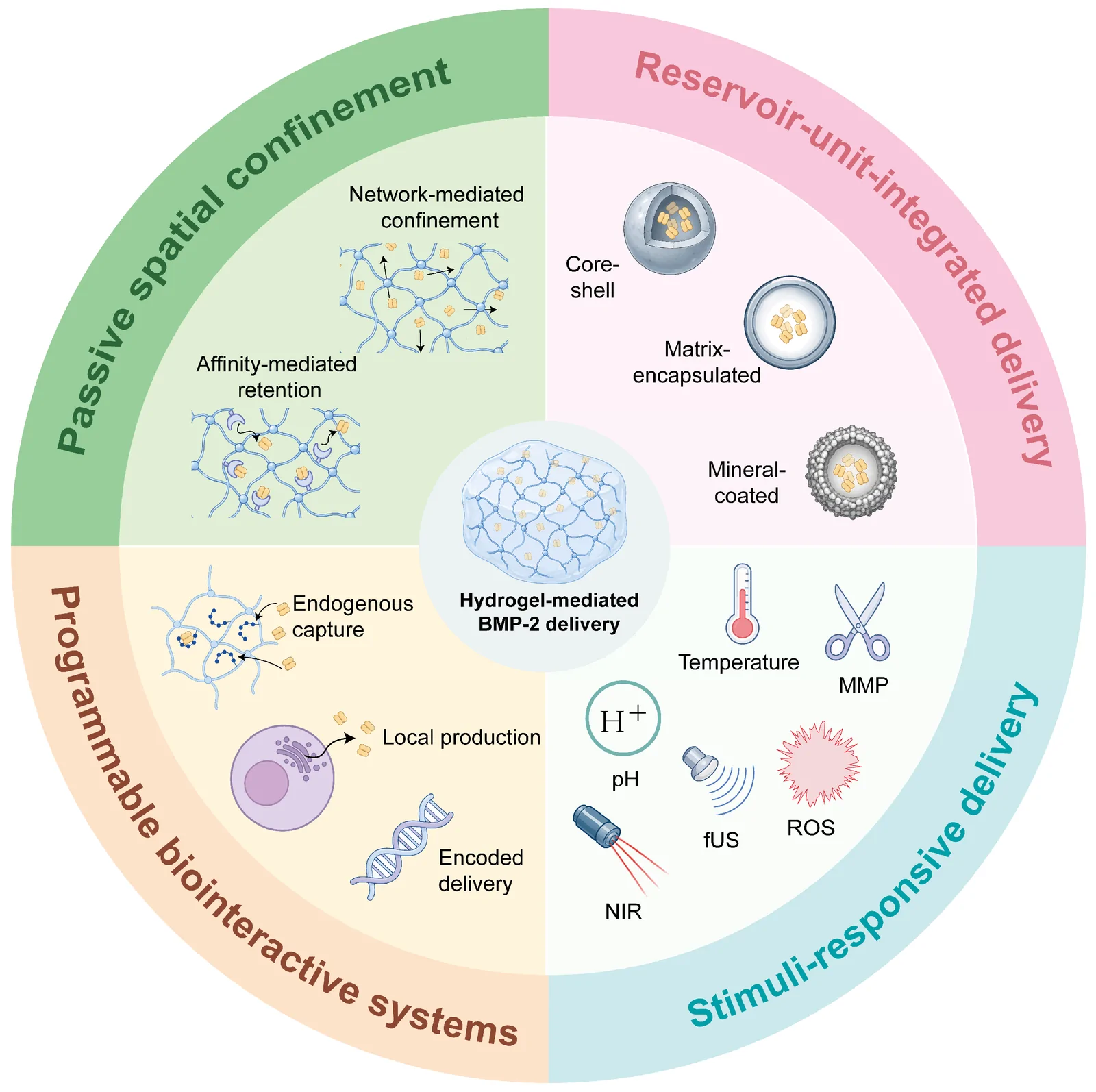
Mechanism-based classification of hydrogel platforms for BMP-2 delivery. The platforms are organized according to the principal mode by which the material controls BMP-2 localization, availability, release, or local production: passive spatial confinement, reservoir-unit-integrated delivery, stimuli-responsive delivery, and programmable biointeractive systems. Representative implementations include network-mediated confinement, affinity retention, core-shell or matrix-encapsulated reservoirs, mineral-coated reservoirs, endogenous BMP-2 capture, encoded delivery, local biological production, and stimulus-mediated regulation by temperature, matrix metalloproteinases (MMPs), pH, reactive oxygen species (ROS), near-infrared irradiation (NIR), and focused ultrasound (fUS). The categories indicate dominant delivery logic rather than comparative therapeutic superiority.

**Figure 3:**
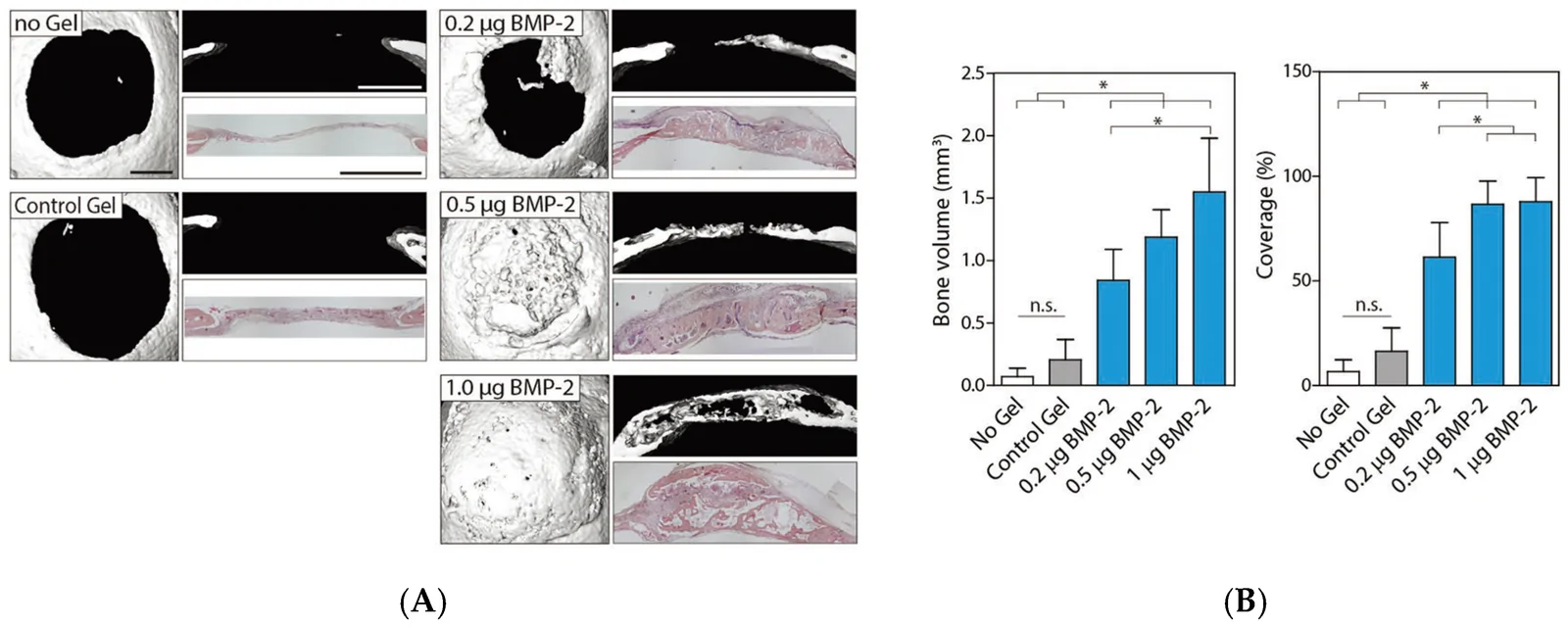
Dose-dependent bone regeneration induced by hydrogel-delivered BMP-2. Representative micro-CT reconstructions, hematoxylin and eosin (H&E)-stained sections, and quantitative assessments of bone formation in 4 mm critical-sized mouse calvarial defects at 4 weeks after delivery of different doses of soluble BMP-2. Data are presented as mean ± SD for *n* = 6 independent defects. n.s., not significant; * *p* < 0.05 (one-way ANOVA with Tukey-Kramer post hoc test). Adapted from Lienemann et al. [39] under the Creative Commons Attribution 4.0 International License (CC BY 4.0; https://creativecommons.org/licenses/by/4.0/). Original panels (**A**,**B**) were selected and cropped; no scientific content within these panels was altered.

**Figure 4:**
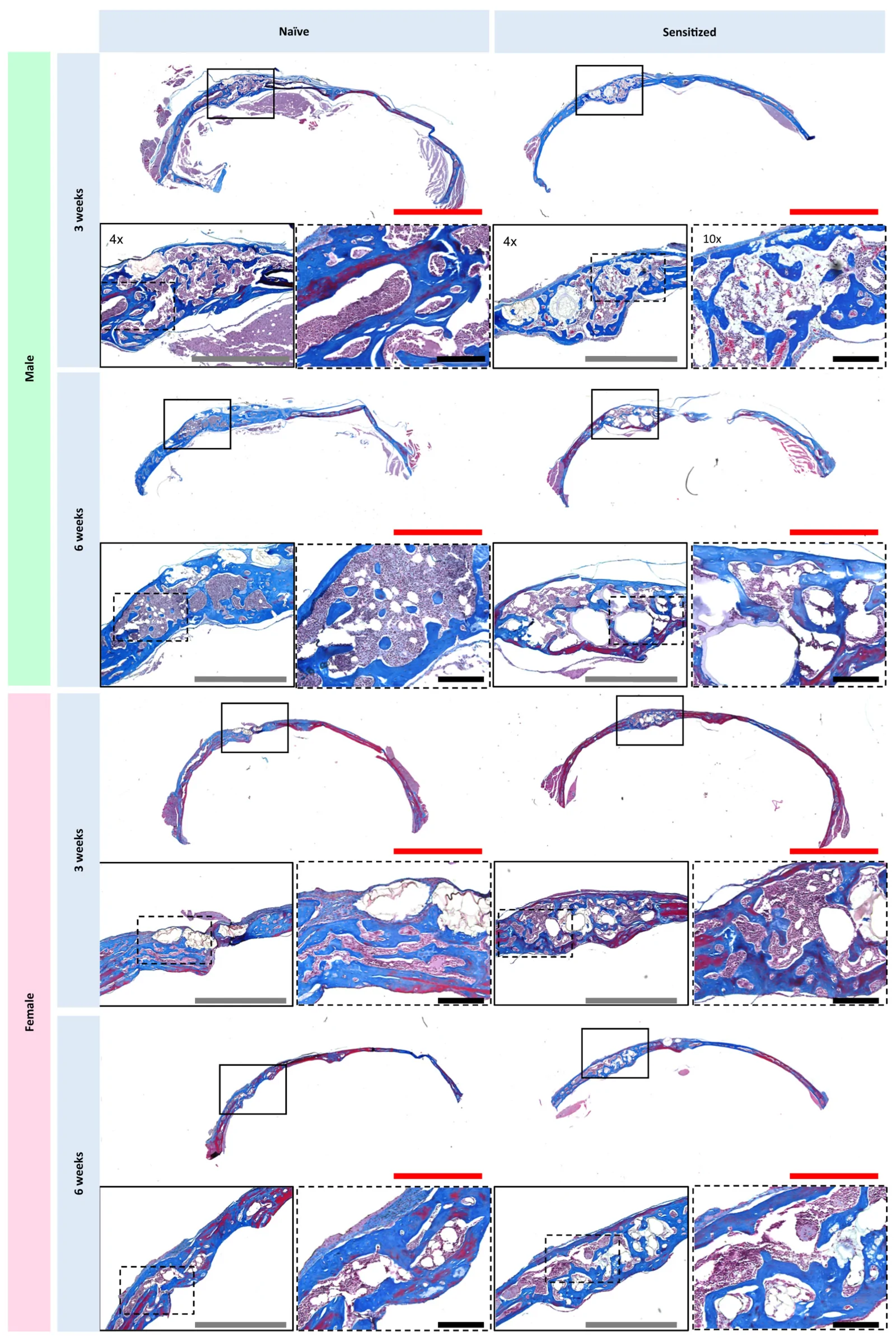
PEG sensitization was associated with disorganized collagen architecture in calvarial defects treated with PEG MAP + BMP-2. Masson’s trichrome staining of naive and PEG-sensitized male (top) and female (bottom) mice at 3 and 6 weeks after implantation showed that the difference in collagen organization was most evident in males. The entire calvaria, including the defect site and contralateral uninjured bone, is shown (scale bar = 3 mm). Solid-line boxes indicate defect sites magnified at ×4 (scale bar = 1 mm), and dashed-line boxes indicate regions further magnified at ×10 (scale bar = 200 μm). Tissue samples from *n* = 4 mice per treatment group were analyzed. Reproduced from [106] under the Creative Commons Attribution 4.0 International License (CC BY 4.0; https://creativecommons.org/licenses/by/4.0/).

**Table 1:** Representative BMP-2-delivering hydrogel systems and their dominant control mechanisms.

Mechanistic Class	Representative Hydrogel System	BMP-2 Modality	Dominant Delivery Mechanism	Release/Production/ Presentation Characteristics	Evaluation Model and Reference
Passive spatial confinement systems	Transglutaminase-crosslinked PEG hydrogel with tunable stiffness, RGD content, and degradable crosslinks	rhBMP-2 protein	Physical incorporation of BMP-2 within a continuous PEG network; stiffness, crosslinking, and degradation jointly alter the diffusion path	All formulations showed first-day burst release; 7-day release varied with network parameters, and released protein retained ALP-inducing activity	In vitro release/C2C12; rat 8 mm calvarial defect; [49]
Passive spatial confinement systems	Fucoidan-functionalized deacetylated β-chitin self-gelling system	rhBMP-2 protein	Affinity binding between a sulfated-polysaccharide interface and BMP-2 reduces early diffusion	Cumulative BMP-2 release: SDGC, approximately 72.5% at day 4; fucoidan-functionalized FD-SDGC, approximately 20.3% at day 4 and 37.1% at day 14	In vitro release/cells; rat 5 mm calvarial defect; [55]
Passive spatial confinement systems	PEG-silk hydrogel containing PLA or PLGA particles	rhBMP-2 protein	Particle incorporation modifies matrix hydrophobicity and stability to regulate BMP-2 retention and release	PLA incorporation reduces the 24 h burst relative to PEG-silk alone; cumulative release evaluated over 61 days	In vitro release and bioactivity; rat 8 mm calvarial defects; [53]
Passive spatial confinement systems	Injectable self-healing CMCS/PEG/HS hydrogel	rhBMP-2 protein	Electrostatic affinity binding by negatively charged HS and degradation of the continuous network jointly control BMP-2 retention and release	Release proceeds mainly with matrix degradation rather than solely through a concentration gradient	In vitro release/cells; rat bilateral 3 mm calvarial defects; [56]
Passive spatial confinement systems	D-BMP2b peptide amphiphile/supramolecular PA gel composite	rhBMP-2 protein	D-BMP2b provides selective binding sites and forms a peptide-mediated affinity-retention interface	Supports sustained local presentation; the low-dose comparison simultaneously changes dose and carrier and therefore cannot be attributed solely to the hydrogel	In vitro BMSCs; rabbit 6 mm calvarial defect and rat 7 × 4 × 3 mm alveolar cleft; [58]
Reservoir-unit-integrated delivery systems	Ni-NTA microparticle-integrated gelatin-transglutaminase-tannic acid hydrogel	His_6_-tagged T4 lysozyme-BMP-2 fusion protein	His_6_-Ni^2+^-NTA coordination directionally anchors the fusion protein to microparticles; the hydrogel immobilizes the particles and provides a second diffusion barrier	Cumulative His6-T4L-BMP2 release in PBS at 37 °C reached approximately 4.5% by day 37 with microparticles versus 9% without, with accelerated release after day 20 in the latter group.	In vitro release; rat 4.5 mm calvarial defect and mouse subcutaneous ectopic model; [61]
Reservoir-unit-integrated delivery systems	MgFe-LDH nanosheets in a chitosan/silk fibroin hydrogel co-delivering PDGF-BB	BMP-2 protein	Chondroitin sulfate-mediated electrostatic immobilization of BMP-2 on LDH nanosheets	In vitro cumulative release: approximately 74.51% BMP-2 over 35 days and 80.03% PDGF-BB within 7 days	In vitro release and cellular assays; rabbit calvarial defects; [64]
Reservoir-unit-integrated delivery systems	BMP-2/VEGF-loaded PLGA nanoparticles spatially integrated into bilayer GelMA	rhBMP-2 and VEGF proteins in PLGA nanoparticles	PLGA nanoparticles provide storage and degradation control, while distinct GelMA layers provide spatial compartmentalization and subsequent diffusion paths	Four-week cumulative BMP-2 release was 48.36% versus 97.2% with direct physical encapsulation	In vitro cells; bilateral 5 mm calvarial defects in male BALB/c mice; [65]
Reservoir-unit-integrated delivery systems	BMP-2-loaded 4armPEG-OPA/gelatin hydrogel infused into a 3D-printed porous PLGA scaffold	BMP-2 protein	The hydrogel acts as a protein reservoir within scaffold pores, whereas the PLGA scaffold provides long-term spatial and mechanical support	Approximately 40% was released during the first 5 days, followed by a slower phase; cumulative release reached 72.4 ± 0.53%, and the hydrogel degraded over approximately 3 weeks	In vitro release/osteogenesis; rabbit femoral condylar defect model; [66]
Reservoir-unit-integrated delivery systems	Bilayer oxidized sodium alginate-carboxymethyl chitosan hydrogel microspheres	BMP-2 protein	The inner layer electrostatically adsorbs BMP-2, while the outer porous polyelectrolyte membrane provides a second diffusion barrier	Approximately 50% was released during the first 5 days; approximately 35% remained in the microspheres at day 10, and release remained detectable at day 20	In vitro rat BMSCs; nude-mouse subcutaneous ectopic model combined with calcium phosphate cement; [67]
Stimuli-responsive delivery systems	Thermosensitive BOX hydrogel	rhBMP-2 protein	Body temperature triggers a sol-gel transition and forms a local protein reservoir in situ	Sustained release accompanies hydrogel degradation after in situ gelation; this is temperature-triggered depot formation rather than pathological-signal-triggered release	In vitro release; rabbit 10 mm femoral defect; [68]
Stimuli-responsive delivery systems	MMP-sensitive PEG nanocomposite hydrogel	Covalently tethered rhBMP-2 and rhBMP-9	rhBMP-2 is attached to PEG chains through a stable tether; the MMP-sensitive peptide serves only as an independent network crosslink	MMP cleavage opens or degrades the network and alters exposure of immobilized BMP-2; BMP-2 remains attached to PEG chains or degradation fragments rather than being released as free protein	BMP-specific reporter assay and encapsulated human MSCs; [69]
Stimuli-responsive delivery systems	PDS hydrogel integrating CaCO_3_-mineralized ZIF-90@BMP-2 and DFO	rhBMP-2 in CaCO_3_-mineralized ZIF-90	Acidic conditions promote stepwise disintegration of the mineral layer and ZIF-90, regulating reservoir stability	In an accelerated in vitro release experiment, DFO peaked at approximately 41 h, whereas BMP-2 release continued to approximately 211 h, producing an approximately 170 h temporal separation	In vitro release; rat 4 mm segmental tibial defect; [70]
Stimuli-responsive delivery systems	PVA-TSPBA boronate-ester hydrogel integrating EGCG nanoparticles and BMP-2@TA-ZIF-8	BMP-2 in TA-modified ZIF-8	H_2_O_2_ alters the boronate-ester network and increases reservoir exposure, while ZIF-8 continues to restrict BMP-2 diffusion	At day 25, cumulative BMP-2 release was 52.9 ± 1.6% in PBS and 65.8 ± 2.1% in H_2_O_2_, supporting condition-dependent ROS-responsive release	In vitro release/cells; ovariectomized rat 5 mm calvarial defect; [41]
Stimuli-responsive delivery systems	HBC thermosensitive hydrogel containing aspirin and PDA-modified Mg-CaCO_3_/BMP-2 microspheres	BMP-2 protein in PDA-modified Mg-CaCO_3_ microspheres	NIR photothermal conversion changes the state of the microspheres and surrounding matrix and promotes BMP-2 release	Fourteen-day cumulative release increased from 37.66 ± 3.39% without NIR to 49.16 ± 2.09% with NIR	In vitro release; rat bilateral 5 mm calvarial defects; [74]
Stimuli-responsive delivery systems	Diels-Alder-crosslinked PEG hydrogel	BMP-2 protein	fUS triggers retro-Diels-Alder de-crosslinking and noninvasively opens release pathways	BMP-2 release increases with ultrasound exposure duration and number; released protein retains ALP- and mineralization-inducing activity	In vitro release and BMSC evaluation; no in vivo bone-defect validation; [75]
Programmable biointeractive systems	CP05-linked *Bmp2* mRNA-enriched engineered exosomes in GelMA hydrogel	*Bmp2* mRNA-enriched engineered exosomes	Hydrogel-released exosomes deliver Bmp2 mRNA for cellular BMP-2 production	The controlled object is local retention and release of encoded information; BMP-2 is produced intracellularly in recipient cells	In vitro uptake/expression; rat calvarial defect model; [77]
Programmable biointeractive systems	NO-sensing engineered bacteria encapsulated in GelMA microspheres and embedded in HAMA hydrogel	In situ BMP-2 production by NO-responsive engineered bacteria	Local NO activates bacterial *BMP-2* synthesis, while the hydrogel confines the bacteria and organizes local signal output	NO-responsive bacterial BMP-2 production rather than release of preloaded protein.	In vitro secretion; mouse cranial and femoral bone-defect models; [45]
Programmable biointeractive systems	BBP-functionalized tetrahedral framework nucleic acid-modified HAMA hydrogel	Endogenous BMP-2 capture	Multivalent display of BMP-2-binding peptides on tFNA enriches and presents endogenous BMP-2 generated during repair	Modifies local capture and spatial availability of endogenous BMP-2 and is not a preloaded-protein controlled-release system	In vitro binding; bone-defect model; [46]
Programmable biointeractive systems	B2A peptide-functionalized HAMA/NHAP-aldehyde-modified HAMA gradient hydrogel	Endogenous BMP-2 capture	NHAP-aldehyde-modified HAMA captures endogenous BMP-2, while grafted B2A promotes osteogenesis in the bone layer and chondrogenesis in the cartilage layer.	Provides spatial compartmentalization of endogenous BMP-2; the osteochondral repair setting lies at the boundary of this review’s scope	Osteochondral gradient-repair model; [80]

Abbreviations: 3D, three-dimensional; 4armPEG, four-arm poly(ethylene glycol); ALP, alkaline phosphatase; BBP, BMP-2-binding peptide; BMSC, bone marrow-derived mesenchymal stem cell; BMP-2, bone morphogenetic protein-2; BOX, bis(2-oxazoline)-linked mPEG-PLGA copolymer; CaCO_3_, calcium carbonate; CMCS, carboxymethyl chitosan; DFO, deferoxamine; EGCG, epigallocatechin gallate; fUS, focused ultrasound; GelMA, gelatin methacryloyl; HAMA, hyaluronic acid methacryloyl; HBC, hydroxybutyl chitosan; His_6_, hexahistidine tag; HS, heparan sulfate; H_2_O_2_, hydrogen peroxide; LDH, layered double hydroxide; MMP, matrix metalloproteinase; mRNA, messenger RNA; MSC, mesenchymal stem cell; NHAP, nanohydroxyapatite; Ni-NTA, nickel-chelated nitrilotriacetic acid; NIR, near-infrared; NO, nitric oxide; OPA, o-phthalaldehyde; PA, peptide amphiphile; PBS, phosphate-buffered saline; PDA, polydopamine; PDGF-BB, platelet-derived growth factor-BB; PDS, polyacrylamide/polydopamine/silk fibroin network; PEG, poly(ethylene glycol); PLA, poly(lactic acid); PLGA, poly(lactic-co-glycolic acid); PVA, poly(vinyl alcohol); RGD, arginine-glycine-aspartic acid; rhBMP-2, recombinant human bone morphogenetic protein-2; rhBMP-9, recombinant human bone morphogenetic protein-9; ROS, reactive oxygen species; tFNA, tetrahedral framework nucleic acid; T4L, T4 lysozyme; TSPBA, ROS-responsive boronic acid crosslinker; VEGF, vascular endothelial growth factor; ZIF, zeolitic imidazolate framework. *BMP2* and *Bmp2* are human and rodent gene symbols, respectively; BMP-2 denotes the protein. B2A, CP05, and D-BMP2b are source-defined peptides; C2C12 is a mouse myoblast line.

**Table 2:** Qualitative comparison of BMP-2 modalities for hydrogel-based bone regeneration.

BMP-2 Modality	Onset	Duration	Administered Input/ Dose Definition	Controllability	Safety Considerations
Protein [12,15,69,75]	Upon local release or cell-accessible presentation	Depends on carrier retention/release and protein persistence	BMP-2 protein mass per implant or defect	Tunable through binding, degradation, and formulation-specific release triggers	Dose-, carrier-, and site-dependent adverse effects, including off-target bone formation
pDNA [76]	After cellular uptake, nuclear entry, transcription, and translation	Depends on plasmid delivery, persistence, and transcriptional activity	Plasmid amount; carrier input specified separately	Carrier design regulates delivery but does not directly prescribe subsequent protein output	Transfection-related effects and long-term gene-delivery safety require consideration
mRNA [77,78]	After cytoplasmic delivery and translation	Generally transient; depends on mRNA stability and the duration of carrier-mediated delivery	mRNA amount; EV quantity and RNA cargo distinguished for EV formulations	Carrier design regulates delivery and retention; protein output is not directly equivalent to mRNA input	Formulation-specific cytocompatibility, distribution, and longer-term safety require evaluation
Engineered cells [79]	Through secretion by viable, premodified cells	Depends on cell survival and maintenance of expression	Cell number, or cell density with construct volume	Cell input and placement are specified; secretion depends on cell persistence and expression	Cell persistence, immune compatibility, and prolonged secretion require consideration
Endogenous BMP-2 capture [46]	Upon binding of locally available host BMP-2	Depends on host BMP-2 supply and persistence of binding sites	Scaffold/ligand input; no exogenous BMP-2 dose	Binding can be tuned through ligand organization; host production is not directly controlled	Scaffold/ligand biocompatibility and effects on endogenous signaling require evaluation

Abbreviations: pDNA, plasmid DNA; mRNA, messenger RNA; EV, extracellular vesicle.

**Table 3:** Comparison of representative in vivo and clinically relevant evidence for BMP-2-delivering hydrogels.

Study and Platform	Model and Host Condition	Key Dose/Comparator Design	Main Regenerative/Spatial Outcomes	Supported Inference	Main Limitations and Reference
HMPs for enhanced local retention	Rat 8 mm critical-size femoral defect; healthy model	Same-dose comparison (2.5 μg/defect) of BMP-2 alone and HMP + BMP-2, including blank HMPs	At 12 weeks, bridging occurred in 5/5 and 3/5 defects, respectively; overall bone formation was greater with BMP-2 alone	Slower in vitro release and greater early in vivo retention did not improve healing, directly challenging the assumption that greater retention necessarily improves efficacy	Small sample size; no dose gradient or dedicated spatial endpoint; [33]
Silk hydrogel within perforated PCL nanofiber mesh tubes	Healthy rats; plate-stabilized 8 mm femoral segmental defects	1% versus 2% silk; 5 μg BMP-2/defect; matched BMP-2-free controls	At 12 weeks, bridging in 4/7 and 4/5 BMP-2-treated defects versus 0/5 and 0/4 controls; pooled BMP-2 groups showed greater bone volume, torsional stiffness, and maximum torque	Different in vitro release profiles did not yield significant differences in bone repair between silk concentrations	No in vivo release measurements; incomplete bridging; attrition reduced statistical power; [96]
HMP-mediated regulation of bone distribution inside and outside the defect	Rat 8 mm femoral defect; healthy model	Same-dose comparison (30 μg/defect) using 0, 0.1, and 1 mg HMP	At 4 weeks, the fraction of bone outside the defect decreased from 65.8% to 39.4% and 44.8%; bridging, mineral density, and torsional load were similar at 12 weeks	A delivery-platform benefit can appear as improved spatial localization rather than increased total bone or mechanical performance	Bone outside the defect in this model is not equivalent to clinically symptomatic ectopic ossification; [15]
Rapid click-crosslinked PEG hydrogel	Mouse 5 mm calvarial defect; healthy model	Same-dose comparison (1 μg/defect) of PEG hydrogel and collagen carrier, including BMP-2-free controls	Defect closure was similar between carriers; more bone formed outside the defect with collagen, whereas the PEG group did not differ significantly from the BMP-2-free control	Rapid gelation and physical confinement may limit the spatial extent of bone formation without requiring prolonged release	Approximately 87% of BMP-2 was released from PEG in vitro within 2 days; collagen release was not measured in parallel, and some sample reporting was inconsistent; [95]
D-BMP2b affinity supramolecular gel	Rabbit 6 mm calvarial defect and rat alveolar cleft; healthy models	Rabbit: same-dose carrier comparison; rat: 1.5-μg affinity gel versus 15-μg collagen	The affinity gel achieved more complete repair at the same dose in rabbits; low-dose gel and high-dose collagen produced similar bone-regeneration proportions in rats	Provides signals for both carrier effects and low-dose feasibility, suggesting that dose and carrier jointly determine the repair/inflammation balance	The low- versus high-dose comparison also changes the carrier and therefore cannot establish strict tenfold dose sparing or noninferiority; [58]
Thermosensitive BOX hydrogel dose gradient	Rabbit 10 mm femoral defect; healthy model	Within-platform dose gradient of 0, 5, 10, and 20 μg/defect, including blank hydrogel and autograft	Twelve-week healing rates were 0/6, 2/6, 4/6, and 6/6; mechanical performance of the 20-μg group approached or exceeded that of autograft	Establishes a within-platform relationship among dose, bridging, and mechanical performance and helps define an effective dose range	No same-dose standard carrier; therefore, dose sparing relative to another carrier cannot be demonstrated; [68]
HAp/β-TCP/hydrogel composite carrier	Rat caudal interbody fusion; healthy model	Dose-matched collagen versus composite hydrogel at 3 and 10 μg/fusion site	Fusion rates were 50%/62.5% with collagen and 87.5% at both doses with the composite carrier; non-target bone formation occurred in 50%/87.5% and 12.5%/25%, respectively	At the same dose, the carrier can alter the balance between fusion efficacy and bone formation outside the target region	Release was not measured at either dose, precluding attribution of observed differences to sustained release; [93]
PEG hydrogel with tunable network stiffness, RGD, and MMP degradability	Rat 8 mm calvarial defect; healthy model	Same-dose comparison (5 μg/defect) of network stiffness, RGD, and MMP-sensitivity combinations	The amount and intradefect distribution of bone varied with parameter combinations; no single parameter determined the outcome monotonically	Material parameters have conditional windows, and network degradation, tissue entry, and BMP-2 availability must be jointly matched	Multivariable interactions limit isolation of individual contributions; bone outside the defect was not evaluated; [49]
HAp/β-TCP/hydrogel in miniature-pig OLIF	50 kg miniature-pig lumbar fusion with PEEK cage and internal fixation	Within-platform dose gradient of 0, 500, and 1000 μg/fusion site; evaluated at 8 and 16 weeks	Fusion scores did not differ significantly between 500 and 1000 μg; 1000 μg produced canal-directed osteophytes, fibrosis, and inflammation	Provides rare large-animal evidence of a dose-fusion-spatial-risk window; increasing the dose did not further improve fusion	No standard carrier or autograft comparator. The statistical unit and sample allocation were not clearly reported. Mechanical, neurological, and long-term safety outcomes were not evaluated; [94]
ROS-responsive EGCG/BMP-2 composite hydrogel	Ovariectomized rat bilateral 5 mm calvarial defects; osteoporosis	BMP-2-only module, EGCG-only module, and complete combined platform	The complete platform achieved 55.1 ± 3.1% new bone volume fraction at 6 weeks, with vascular- and immune-related changes	Microenvironmental regulation and BMP-2 delivery can jointly expand the net repair window in an abnormal bone-remodeling environment	Main quantitative analyses used *n* = 3; EGCG, ZIF-8, and the responsive network changed simultaneously, so the benefit cannot be attributed entirely to BMP-2 release; [41]
Acid-responsive MPN/BMP-2 microenvironment-regulating hydrogel	18-month-old ovariectomized mouse femoral defect; osteoporosis/aging	Comparison including a BMP-2-only module and the complete combined platform	The BMP-2-only module produced the highest Tb.N and lowest Tb.Sp, whereas the combined group had higher BV/TV and BMD	Combined regulation was not superior to BMP-2 alone for every bone parameter; bone amount, density, and microarchitecture should be evaluated separately	Divergent endpoint directions do not support claims of universal synergy or comprehensive superiority; [40]
Modular IL-10/BMP-2 hydrogel	Rat calvarial defect with glycemic variability in diabetes	Blank, IL-10-only, BMP-2-only, and combined-delivery groups	The combined group achieved 97.2% bone-connection ratio at 8 weeks and greater bone volume than either single-module group, with improvement in early inflammatory/oxidative-stress indicators	Relatively complete component controls support a joint effect of diabetes-environment regulation and BMP-2 on structural repair	Associated biomarker changes alone do not establish that inflammatory modulation mediated the therapeutic benefit; [99]
PEG-4MAL hydrogel co-delivering BMP-2 and lysostaphin (LST)	*Staphylococcus aureus*-infected mouse 2.5 mm segmental radial defect	Sterile BMP-2, untreated infection, infection + LST, gentamicin, and other groups; 100 ng BMP-2 plus 1 U LST/defect	Untreated infected defects formed no new bone at 8 weeks; LST co-delivery restored new bone, bridging, and torsional performance	Establishes a continuous evidence chain linking infection control, restoration of the inflammatory environment, structural bridging, and mechanical recovery	Single bacterial strain and mouse model; absence of a detected difference cannot be interpreted as equivalence or extrapolated to chronic polymicrobial osteomyelitis; [32]
MAP hydrogel delivering BMP-2 in PEG-sensitized hosts	Naive and PEG-KLH-sensitized mice with 2.7 mm calvarial defects	Same hydrogel and dose (500 ng/defect), with host sensitization as the primary variable; empty-defect, MAP, and MAP + BMP-2 controls	Sensitized animals showed greater bone volume and collagen area, but the new bone was more porous and collagen was more disorganized	The same BMP-2 hydrogel can produce increased bone quantity without corresponding improvement in tissue quality when host material immunity differs	Single artificial sensitization model; findings cannot be generalized to all PEG materials or human populations; [106]
*Escherichia coli*-derived recombinant human BMP-2/β-TCP hydrogel composite product for ALIF	Twenty adults with spinal deformity undergoing L5-S1 ALIF; prospective single-arm pilot	No concurrent control; 3 mg/fusion level; composite product used during complex corrective surgery	Fusion was observed in 13/19 patients at 6 months and 18/18 evaluable patients at 12 months; 5/20 patients experienced 10 adverse events	Supports one-year clinical feasibility and a fusion signal for a specific product-procedure combination	No control, small sample, and loss to follow-up; cannot establish relative efficacy, the independent hydrogel contribution, or definitive safety; [117]
*Escherichia coli*-derived recombinant human BMP-2/HAp/β-TCP-poloxamer hydrogel for PLIF	254 patients with degenerative lumbar disease; 357 fusion levels; retrospective comparison	Composite product (maximum 0.5 mg/fusion level) plus local autograft versus local autograft alone	Dynamic-radiograph fusion rates were 92.31% versus 82.35%; CT fusion rates were 93.21% versus 88.24% without a significant difference; functional outcomes showed no between-group benefit	Provides a real-world active-comparator signal, but the apparent efficacy advantage was not consistent across CT and patient-reported outcomes	Nonrandomized; multicomponent product and autograft; nested spinal levels and one-year follow-up; findings cannot be attributed specifically to the hydrogel; [118]

Abbreviations: ALIF, anterior lumbar interbody fusion; BMD, bone mineral density; BMP-2, bone morphogenetic protein-2; BOX, bis(2-oxazoline)-linked mPEG-PLGA copolymer; BV/TV, bone volume/total volume; CT, computed tomography; D-BMP2b, source-defined BMP-2-binding peptide; EGCG, epigallocatechin gallate; HAp, hydroxyapatite; HMP, heparin methacrylamide microparticle; IL-10, interleukin-10; LST, lysostaphin; MAP, microporous annealed particle; MMP, matrix metalloproteinase; MPN, metal-phenolic network; OLIF, oblique lumbar interbody fusion; PCL, polycaprolactone; PEEK, polyetheretherketone; PEG, poly(ethylene glycol); PEG-4MAL, four-arm maleimide-functionalized poly(ethylene glycol); PEG-KLH, poly(ethylene glycol)-keyhole limpet hemocyanin conjugate; PLIF, posterior lumbar interbody fusion; RGD, arginine-glycine-aspartic acid; ROS, reactive oxygen species; Tb.N, trabecular number; Tb.Sp, trabecular separation; ZIF, zeolitic imidazolate framework; β-TCP, beta-tricalcium phosphate.

## Data Availability

No new data were created or analyzed in this study. Data sharing is not applicable to this article.

## Abbreviations

The following abbreviations are used in this manuscript:

3D	three-dimensional
4armPEG	four-arm poly(ethylene glycol)
AAV	adeno-associated virus
ACS	absorbable collagen sponge
ALIF	anterior lumbar interbody fusion
ALP	alkaline phosphatase
BBP	BMP-2-binding peptide
BMD	bone mineral density
BMSC	bone marrow-derived mesenchymal stem cell
BMP-2	bone morphogenetic protein-2
BOX	bis(2-oxazoline)-linked mPEG-PLGA copolymer
BV/TV	bone volume/total volume
CAT	catalase
CGF	concentrated growth factor
CHO	chinese hamster ovary
CMCS	carboxymethyl chitosan
COX-2	cyclooxygenase-2
CS	chitosan
CT	computed tomography
DFO	deferoxamine
DNA	deoxyribonucleic acid
ECM	extracellular matrix
EGCG	epigallocatechin gallate
EV	extracellular vesicle
fUS	focused ultrasound
GelMA	gelatin methacryloyl
HA	hyaluronic acid
HAMA	hyaluronic acid methacryloyl
HAp	hydroxyapatite
HBC	hydroxybutyl chitosan
HMP	heparin methacrylamide microparticle
HS	heparan sulfate
ID1	inhibitor of DNA binding 1
IL-6	interleukin-6
IL-10	interleukin-10
LNP	lipid nanoparticle
LST	lysostaphin
MAP	microporous annealed particle
MMP	matrix metalloproteinase
MPN	metal-phenolic network
mPEG-PLGA	monomethoxy poly(ethylene glycol)-block-poly(lactic-co-glycolic acid)
mRNA	messenger RNA
MSC	mesenchymal stem cell
NHAP	nanohydroxyapatite
Ni-NTA	nickel-chelated nitrilotriacetic acid
NIR	near-infrared
NO	nitric oxide
OCN	osteocalcin
OLIF	oblique lumbar interbody fusion
OPA	o-phthalaldehyde
OPN	osteopontin
PA	peptide amphiphile
PBS	phosphate-buffered saline
PCL	polycaprolactone
PDA	polydopamine
PDGF-BB	platelet-derived growth factor-BB
PDS	polyacrylamide/polydopamine/silk fibroin network
PEEK	polyetheretherketone
PEG	poly(ethylene glycol)
PEGS-A	PEGylated poly(glycerol sebacate) acrylate
PEG-4MAL	four-arm maleimide-functionalized poly(ethylene glycol)
PEG-KLH	poly(ethylene glycol)-keyhole limpet hemocyanin conjugate
PEG-Mal	maleimide-functionalized poly(ethylene glycol)
PLA	poly(lactic acid)
PLGA	poly(lactic-co-glycolic acid)
PLL	poly-L-lysine
PLIF	posterior lumbar interbody fusion
PRP	platelet-rich plasma
PVA	poly(vinyl alcohol)
RGD	arginine-glycine-aspartic acid
rhBMP-2	recombinant human bone morphogenetic protein-2
rhBMP-9	recombinant human bone morphogenetic protein-9
ROS	reactive oxygen species
Tb.N	trabecular number
Tb.Sp	trabecular separation
tFNA	tetrahedral framework nucleic acid
TRAP	tartrate-resistant acid phosphatase
TSPBA	ROS-responsive boronic acid crosslinker
VEGF	vascular endothelial growth factor
ZIF	zeolitic imidazolate framework
β-TCP	beta-tricalcium phosphate
